# Enhancing structural plasticity of PC12 neurons during differentiation and neurite regeneration with a catalytically inactive mutant version of the zRICH protein

**DOI:** 10.1186/s12868-023-00808-1

**Published:** 2023-08-23

**Authors:** Ashoka C. Bandla, Aditya S. Sheth, Sara M. Zarate, Suraj Uskamalla, Elizabeth C. Hager, Victor A. Villarreal, Maribel González-García, Rafael P. Ballestero

**Affiliations:** 1 Department of Biological and Health Sciences, Texas A&M University-Kingsville, 700 University Blvd, Kingsville, TX 78363 USA; 2 Department of Chemistry, Texas A&M University-Kingsville, Kingsville, TX 78363 USA

**Keywords:** Axon Regeneration, Neuron differentiation, Neuritogenesis, 2’,3’-Cyclic Nucleotide 3’-Phosphodiesterase, Teleost

## Abstract

**Background:**

Studies of the molecular mechanisms of nerve regeneration have led to the discovery of several proteins that are induced during successful nerve regeneration. RICH proteins were identified as proteins induced during the regeneration of the optic nerve of teleost fish. These proteins are 2’,3’-cyclic nucleotide, 3’-phosphodiesterases that can bind to cellular membranes through a carboxy-terminal membrane localization domain. They interact with the tubulin cytoskeleton and are able to enhance neuronal structural plasticity by promoting the formation of neurite branches.

**Results:**

PC12 stable transfectant cells expressing a fusion protein combining a red fluorescent protein with a catalytically inactive mutant version of zebrafish RICH protein were generated. These cells were used as a model to analyze effects of the protein on neuritogenesis. Differentiation experiments showed a 2.9 fold increase in formation of secondary neurites and a 2.4 fold increase in branching points. A 2.2 fold increase in formation of secondary neurites was observed in neurite regeneration assays.

**Conclusions:**

The use of a fluorescent fusion protein facilitated detection of expression levels. Two computer-assisted morphometric analysis methods indicated that the catalytically inactive RICH protein induced the formation of branching points and secondary neurites both during differentiation and neurite regeneration. A procedure based on analysis of random field images provided comparable results to classic neurite tracing methods.

**Graphical Abstract:**

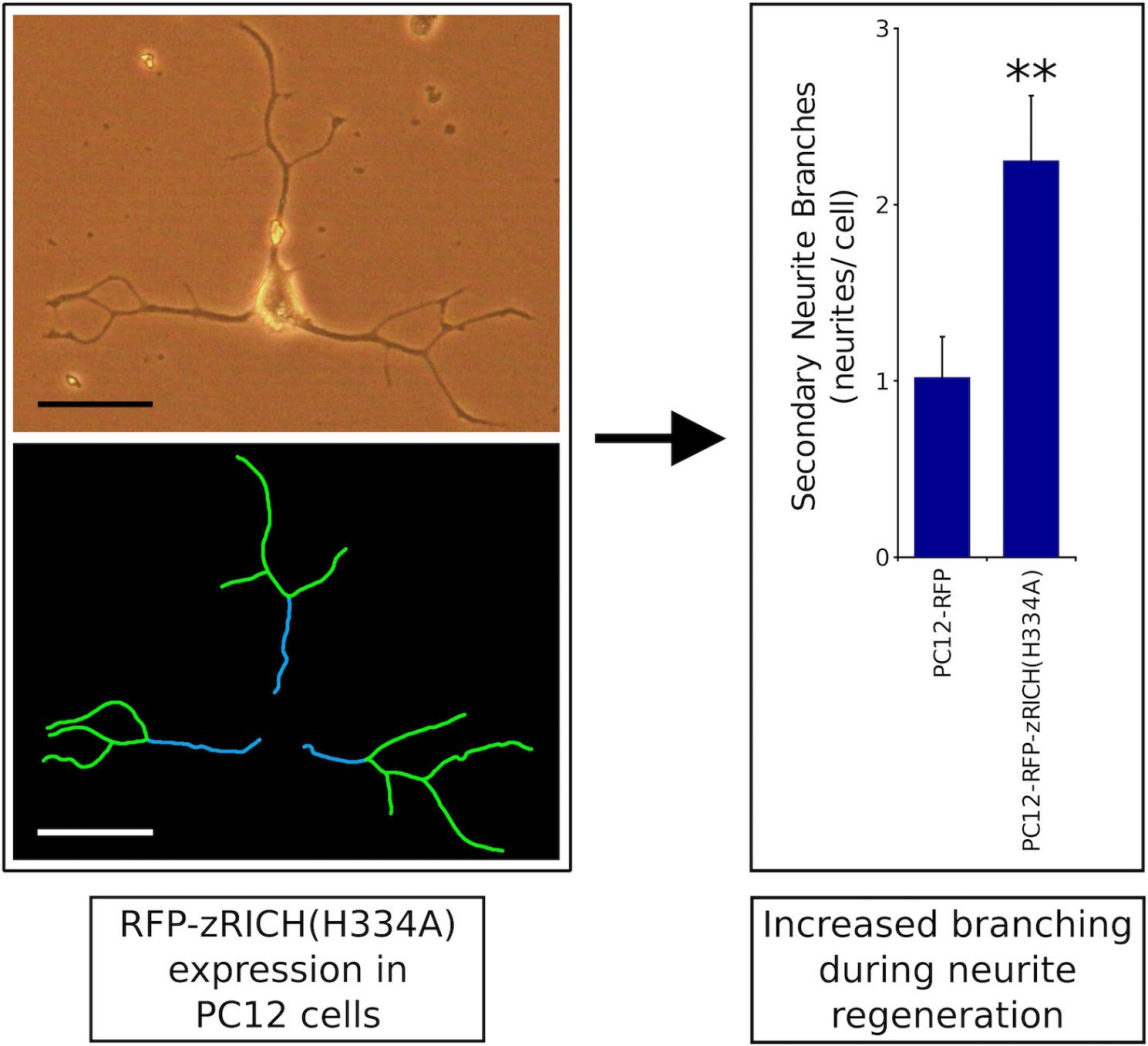

**Supplementary Information:**

The online version contains supplementary material available at 10.1186/s12868-023-00808-1.

## Background

The central nervous system (CNS) of adult mammals exhibits an almost complete absence of spontaneous nerve regeneration [1–4]. This phenomenon has severe biomedical consequences for humans (paralysis conditions derived from spinal cord injury, blindness arising from glaucoma, etc.). These permanent conditions seriously impact the quality of life of the affected individuals, besides the economic burden in health care costs.

Significant levels of axon regeneration occur naturally in the peripheral nervous system (PNS) of mammals, and in both the CNS and PNS of cold-blooded vertebrates [2, 5–11]. Probing the molecular mechanisms that allow nerve regeneration in these systems is of paramount relevance for the development of novel therapies to treat numerous medical conditions derived from damage to the CNS. Studies of these models have led to the discovery of several proteins that are induced during successful nerve regeneration, often referred to as GAPs (Growth Associated Proteins), with the genes encoding them designated as RAGs (Regeneration Associated Genes) [6, 12–16].

One key component required for nerve regeneration is the establishment of an environment supportive of axon regrowth (positive extrinsic factors). Schwann cells in the PNS of mammals have been demonstrated to produce extracellular matrix proteins, cell adhesion molecules and growth factors, all of which promote axon repair [12, 13, 17–19]. Macrophages clear up debris during Wallerian degeneration and also release growth factors that promote regeneration. Conversely, molecules that inhibit growth are produced by both oligodendrocytes and astrocytes in the CNS of mammals (negative extrinsic factors) [20–27].

A second relevant component for successful axon regrowth is the establishment and maintenance of an intrinsic environment in neurons necessary for neurite growth [14, 15, 18, 28–30]. For example, embryonic neurons in the CNS of mammals have high intrinsic capacity for neurite growth, even in the presence of environmental inhibitors [29, 30]. This capacity is reduced in adult neurons, but can be enhanced by supportive environmental cues [3, 14, 18]. Conversely, the neurons of the CNS of cold-blooded vertebrates retain strong capacity for axon regeneration in adults. Cellular support mechanisms in place in the PNS of adult mammals and in both the PNS and CNS of cold-blooded vertebrates result in the induction of the expression of RAGs encoding various intrinsic proteins in neurons, which establish enhanced structural plasticity leading to axonal repair [6, 12, 15, 16, 31–33]. The proteins involved are diverse, including membrane receptors, signal transduction proteins, cytoskeletal proteins, transcription factors, etc.

Diverse cellular and animal model systems have been utilized to characterize the functions and effects of both extrinsic and intrinsic nerve regeneration molecules, contributing to our mechanistic knowledge and the discovery of molecular targets for therapy. Experimental interventions aiming to provide both a supportive environment and to enhance the intrinsic capacity of neurons have demonstrated exciting potential for promoting CNS nerve regeneration in mammals using small rodents as model systems [3, 14, 30, 34–41]. Further testing of molecules that facilitate regeneration may lead to procedures for promoting more potent regeneration that would allow functional recovery in humans in the future.

Our laboratory identified the teleost RICH (regeneration induced CNPase homolog) proteins as homologs of mammalian CNPases (2’,3’-cyclic nucleotide 3’-phosphodiesterases) that are induced during optic nerve regeneration in teleost fish [42–44]. Through mutagenesis experiments, the role of different regions of these proteins in catalysis and subcellular localization was characterized [45]. Interestingly, biochemical and cellular studies suggested that zebrafish RICH can interact with tubulin and enhance structural plasticity of differentiating PC12 neurons [46]. A catalytically inactive version of the protein was discovered to have augmented effects on neurite branching [42, 46]. In this report, this mutant version of zRICH was fused to RFP, allowing for the detection of expression by fluorescent microscopy. Novel, sensitive, and more efficient cellular assays have been developed, utilizing PC12 cells as a model of neuronal differentiation and neurite regeneration. Utilizing these methods, the fusion protein was confirmed to retain the effect of enhancing neuronal plasticity during differentiation, mainly by promoting neurite branching. Additionally, the fusion protein facilitated the detection of a similar effect on neurite regeneration for the first time.

## Results

### Generation of stable PC12 transfectants expressing zRICH(H334A) fused to RFP

**Fig. 1:**
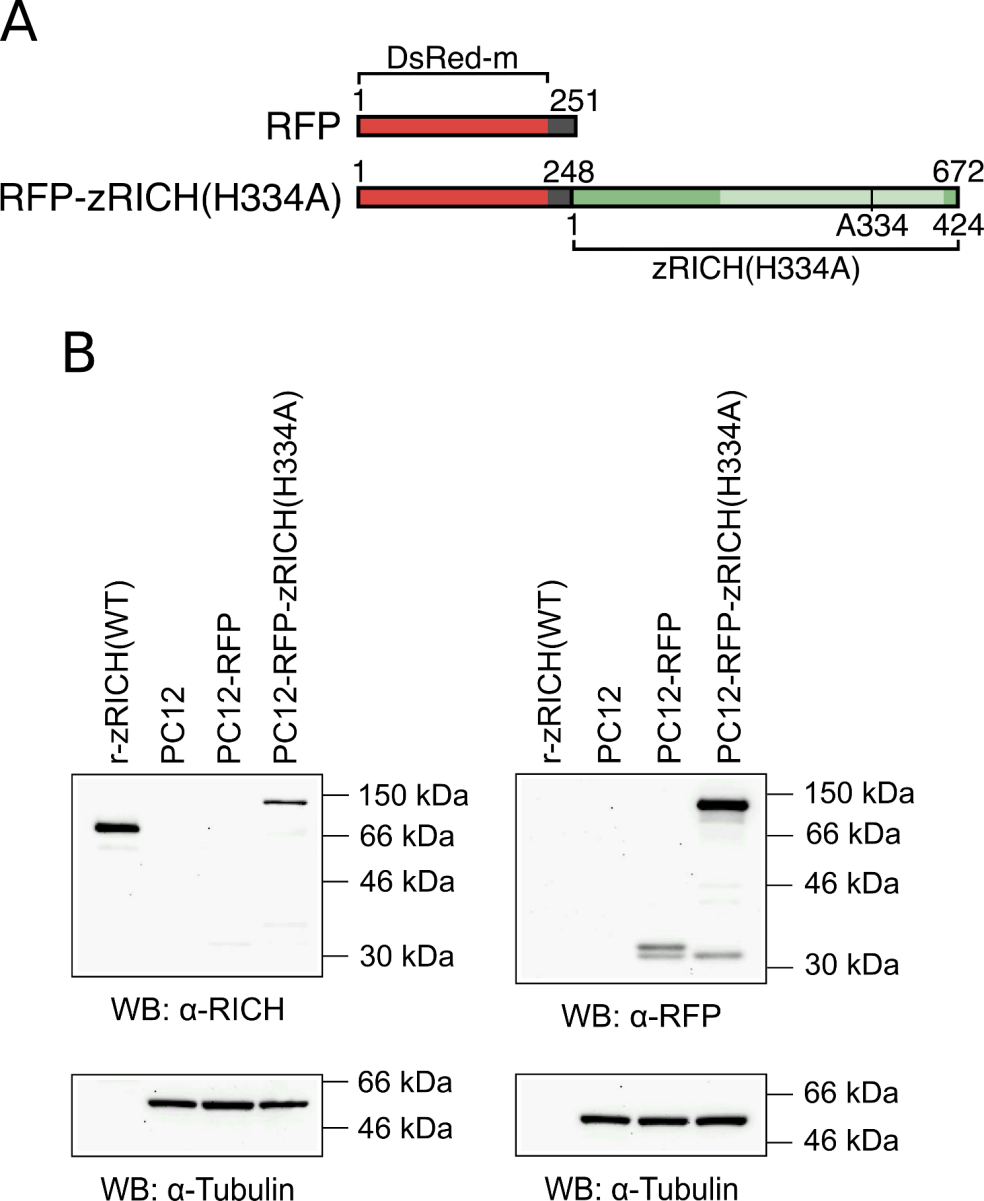
PC12 stable transfectants expressing RFP or RFP-zRICH(H334A) fusion proteins. **A**: Structure of the proteins expressed in PC12 cells. The protein designated as RFP is 251 amino acids long and it is composed of the DsRed-monomer RFP fused to a peptide derived from the polylinker region of the plasmid. The 672 amino acid long fusion protein RFP-zRICH(H334A) consists of the full size 424 amino acid long zRICH(H334A) fused to the amino-terminal 248 amino acid long fragment derived from RFP. The H to A amino acid substitution at position 334 of the zRICH protein portion, affecting a key catalytic site residue within the CNPase homology domain (light green shade) is indicated in the diagram. **B**: Western blot analysis of PC12 stable transfectants. Immunodetection was performed with anti-RICH antibody (top-left membrane) or anti-RFP antibody (top-right membrane). Both anti-RICH and anti-RFP detected the expressed RFP-zRICH(H334A) fusion protein (apparent molecular weight of approximately 100 kDa). The much smaller RFP protein was detected in the lysate from PC12-RFP cells above the 30 kDa size marker. A degradation product detected by anti-RFP just above 30 kDa is present in both of the stable transfectants. Untransfected PC12 cells were used as negative control and 100 ng of recombinant zRICH(WT) was used to confirm antibody specificity and estimate levels of expression of the fusion protein by densitometric analysis (35 ng on the membrane, 0.7 ng/µg of total cell protein). Immunoblots with anti-Tubulin antibody (bottom panels) confirmed that similar amounts of protein lysates from the cells were utilized. Images were cropped to facilitate visualization (using GIMP software). Original unprocessed images of full-length blots are presented in Supplementary Material 7

Previous studies with PC12 cells stably transfected with plasmids expressing wild-type (WT) or mutant versions of zRICH allowed the detection of the effects of these proteins on neuritogenesis [46]. Interestingly, expression of a catalytically inactive mutant version designated as zRICH(H334A) resulted in significant enhancement of neurite branching on PC12 cells differentiated by nerve growth factor (NGF) treatment. The experiments involved fixing the cells for the detection of expression of the protein by immunohistochemistry, followed by manual morphometric analysis. Although successful, the procedure was very labor-intensive and the immunohistochemical detection prevented extension to more ambitious studies. To develop a more efficient assay, a eukaryotic expression plasmid was constructed to fuse a red fluorescent protein (RFP) to the N-terminus of zRICH(H334A). Figure 1A shows a diagram of the 672 amino acid long fusion protein. Both the new plasmid encoding the fusion protein, and the original control plasmid encoding only RFP, were utilized to generate stable PC12 transfectants by selection with G418. Western blot (WB) analysis with extracts from the PC12-RFP or PC12-RFP-zRICH(H334A) stable transfectant cells demonstrated expression of the corresponding proteins (Fig. 1B). The anti-RICH antibody detected both the recombinant histidine-tagged zRICH(WT) protein, used as a control, and the larger RFP-zRICH(H334A) fusion protein in the lysate obtained from the stable transfectant. A WB performed with anti-RFP antibody also detected the same RFP-zRICH(H334A) protein of approximately 100 kDa apparent molecular weight, confirming the identity of the fusion protein. This WB also allowed the detection of the 251 amino acid long RFP molecule (with an apparent molecular weight slightly above 30 kDa) expressed in the lysate obtained from the stable transfectant PC12-RFP cells (containing the integrated original pDsRed-monomer-C1 plasmid). A degradation product slightly smaller than the full-length RFP was observed in both lysates, suggesting partial proteolysis targeting the “linker” region added by the plasmid after the DsRed-m protein sequence (Fig. 1A). As expected, the lysates from the original untransfected PC12 cells did not show any RFP or zRICH protein signal (Fig. 1B). The membranes were stripped and re-probed with anti-Tubulin antibody to confirm that comparable amounts of lysates were utilized in the experiments.

### PC12 stable transfectants expressing RFP fusion proteins as a model to study effects on neuronal differentiation

**Fig. 2:**
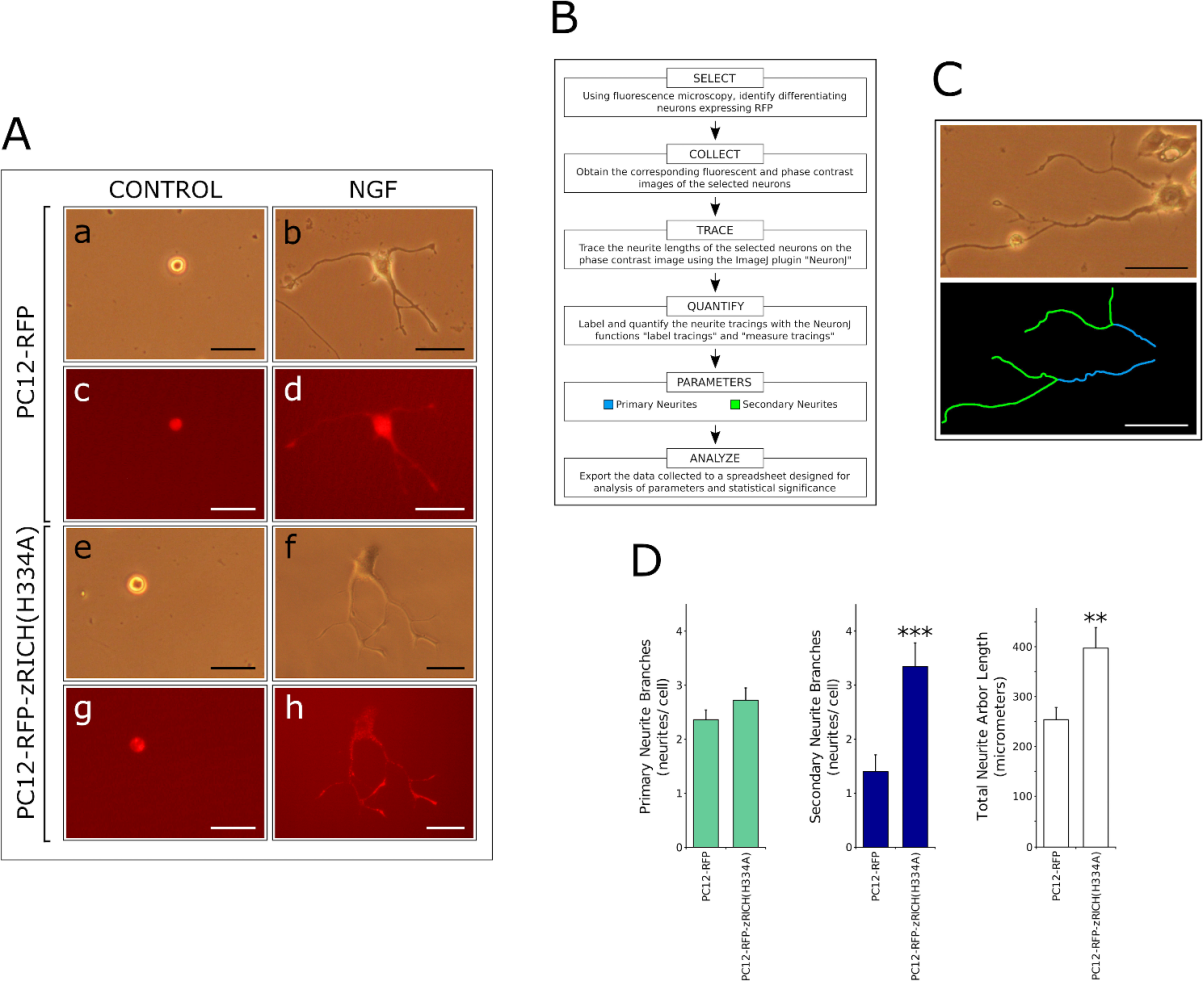
Effects of RFP-zRICH(H334A) expression on PC12 differentiation. **A**: Differentiation assays with PC12 stable transfectants expressing RFP or RFP-zRICH(H334A). PC12-RFP and PC12-RFP-zRICH(H334A) stable transfectant cells were treated with differentiation medium containing 100 ng/ml of NGF. At day 0, cells are spherical under phase contrast microscopy (panels a and e). After 7 days in the presence of NGF, neuronal differentiation is very apparent under phase contrast microscopy by the presence of long neurites several times the length of the cell body (panels b and f). The expression of RFP and RFP-zRICH(H334A) can be detected in the living cells by fluorescence microscopy (panels c, d, g and h). Scale bars represent 50 μm. **B**: Summary of the NeuronJ tracing computer-assisted morphometric analysis procedure. The diagram summarizes the procedure to analyze the neurite arbors in differentiated PC12 cells expressing fluorescent proteins. Neurons that are well isolated and express the fluorescent proteins (detected by fluorescence microscopy) are photographed by both fluorescence and phase contrast microscopy. Neurites are traced in the phase contrast images with the help of NeuronJ to facilitate quantitative analysis. **C**: Example of NeuronJ tracing of a differentiated PC12-RFP-zRICH(H334A) cell. The top panel shows the phase contrast microscopy image and the bottom panel shows the NeuronJ traces. Primary neurite segments arising from the cell body are highlighted in blue and traces of secondary neurite branch segments are highlighted in green. Only neurite segments longer than 1 cell body length were traced. Scale bar represents 50 μm. **D**: Results of morphometric analysis of differentiation assays with the stable transfectants. From left to right, the graphs represent the number of primary neurite branches, the number of secondary neurite segments, and the total neurite arbor length per cell, respectively. The most pronounced effect of the expression of zRICH(H334A) in differentiated PC12 cells was an approximately 2.4 fold increase in secondary neurite segments when compared with cells expressing RFP. A smaller 1.5 fold increase in the total neurite arbor length was observed, probably due mainly to the larger number of secondary neurites. A modest 1.2 fold increase in primary neurites was observed, but it is not statistically significant. The bars show the average ± SEM; n = 55 PC12-RFP and 43 PC12-zRICH(H334A) cells. Statistics: ** t-test, p < 0.01, *** t-test, p < 0.001. The experiment presented is representative of 4 independent assays

The PC12-RFP and PC12-RFP-zRICH(H334A) stable transfectants were used in neuronal differentiation assays by treatment with NGF (Fig. 2A). The transfectants did not show any apparent differences in phenotype under normal growth conditions. The results are consistent with our previous observations in which the expression of WT or mutant versions of zRICH do not result in spontaneous differentiation [45, 46]. In the differentiation assays, the cells were seeded at low densities, and at day 0, before any exposure to NGF, the cells exhibited a spherical shape (Fig. 2A, panels a, e). Fluorescence microscopy allowed the detection of expression of either RFP (Fig. 2A, panel c), or of the RFP-zRICH(H334A) fusion protein (Fig. 2A, panel g) in the living cells. Treatment with NGF triggered neuronal differentiation of both PC12-RFP and PC12-zRICH(H334A) cells, as observed by the extension of neurites, easily detected by phase contrast microscopy (Fig. 2A, panels b and f, which exhibit cells exposed to NGF for 7 days). The same living cells can be analyzed immediately for expression of the protein of interest under fluorescence microscopy (Fig. 2A, panels d, h). The results confirm that these transfectants offer significant advantages in the design of experiments to study the effects of proteins of interest on neuronal differentiation, by facilitating the detection of both morphological characteristics and levels of expression of the proteins in living neurons, in contrast to previous studies requiring fixation and immunohistochemistry to assess the expression of the proteins from transfected genes.

### zRICH(H334A) expression induces neurite branching in PC12 cells

The effects of the expression of the RFP-zRICH(H334A) fusion protein in PC12 cells differentiated to neurons by NGF treatment were studied with a procedure that takes advantage of the detection of expression by fluorescence microscopy (Fig. 2B). Neurons that were well isolated and that expressed high levels of the protein of interest were selected for analysis by computer-assisted tracing analysis with the help of NeuronJ software. Primary neurite segments, originating from the cell body, and secondary neurite branch segments, coming from another neurite, were labeled and quantified. Figure 2C shows an example of the tracing by this procedure of a neuron expressing RFP-zRICH(H334A). Figure 2D shows representative results from a differentiation experiment analyzed by the NeuronJ tracing method, comparing PC12 cells expressing the fusion protein RFP-zRICH(H334A) with control cells expressing RFP (the specific number of neurons analyzed is provided in the legend of Fig. 2D). The expression of RFP-zRICH(H334A) resulted in a statistically significant increase in the number of secondary neurite branches (approximately 2.4 fold in the representative experiment shown in Fig. 2D; 2.9 fold average increase was observed in 4 independent experiments). The results are consistent with our previous observations, where the catalytically inactive version of zRICH showed an enhanced effect on neurite branching [46], and they suggest that the fusion of the RFP moiety did not block the effects of the protein on structural plasticity. A modest induction of primary neurites was consistently observed (approximately 1.2 fold, both for the experiment presented and for the average of 4 independent experiments), but it was not statistically significant for individual experiments. The total neurite arbor length was moderately increased by the expression of the fusion protein (approximately 1.5 fold for both the representative experiment and for the average of 3 independent experiments). This increase is statistically significant, but lesser in magnitude than the increase in the number of secondary branches, suggesting that it is derived mostly from the increase in branching points and not from an increase in the rates of neurite growth. When combined with previous observations [45, 46], the results support that zRICH proteins have specific effects during the later stages of differentiation of PC12 cells.

### Analysis of the effects of zRICH(H334A) on branching points with a procedure that quantitates images of random fields

**Fig. 3:**
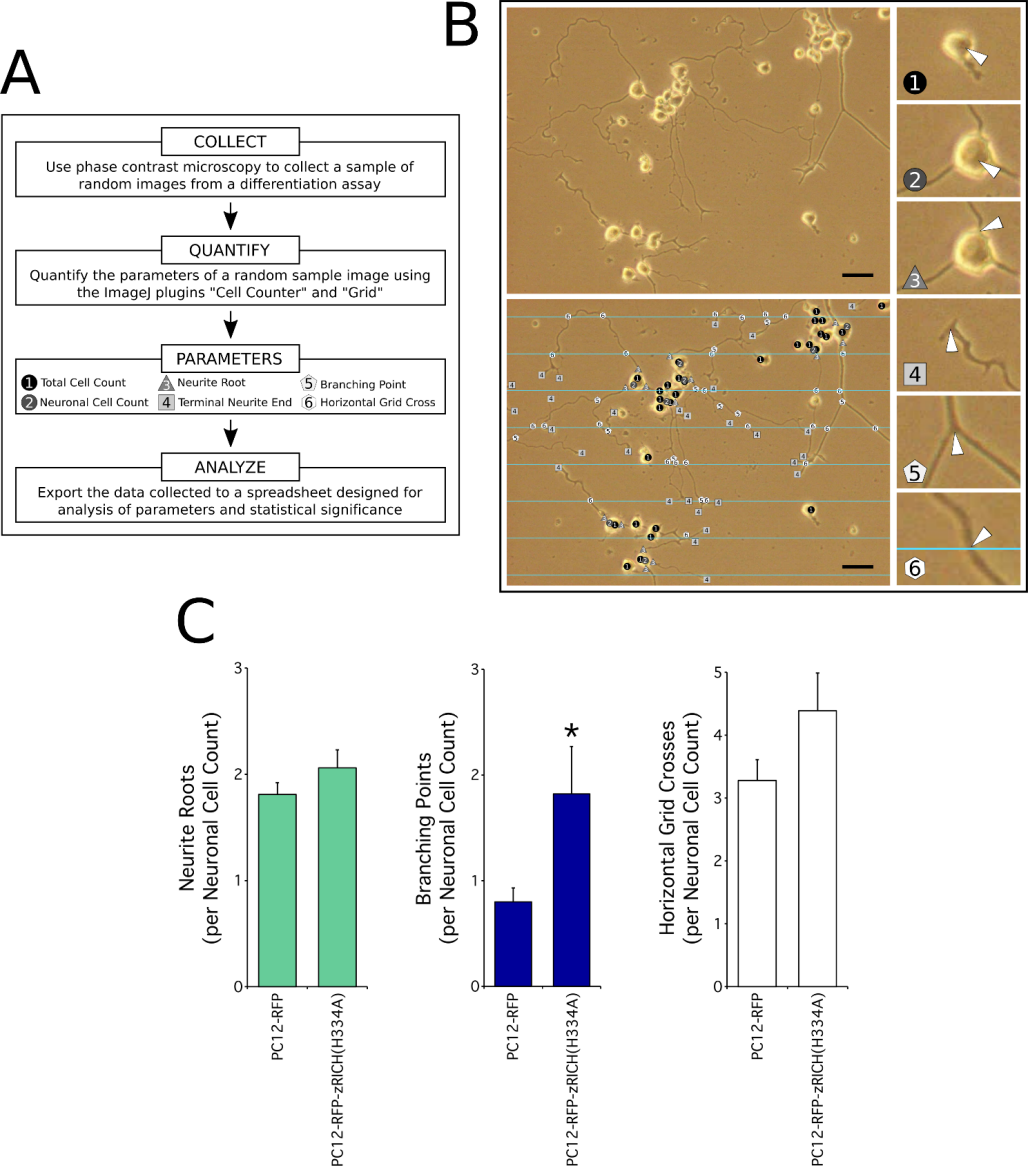
RFP-zRICH(H334A) expression increases branching during PC12 differentiation. **A**: Summary of the computer-assisted random field analysis procedure. The diagram summarizes the morphometric procedure to analyze differentiated PC12 cells in a sample of random field images. The phase contrast images are processed to quantitate various parameters with the help of the “Cell Counter” and “Grid” plugins of ImageJ software. **B**: Example of ImageJ processing of a random field image captured from cultured PC12-RFP-zRICH(H334A) cells treated with NGF for 7 days. The top-left panel shows the original phase contrast microscopy image (scale bar represents 50 μm), and the bottom-left panel shows the image processed with ImageJ to count various parameters. The 6 small images on the right side represent magnified views to illustrate an example of each of the parameters quantitated on the processed image (marked with arrowheads). **C**: Results of morphometric analysis of sets of random field images with the stable transfectants. From left to right, the graphs represent the number of primary neurite roots, the number of branching points, and the number of horizontal grid crosses, normalized by the Neuronal Cell Count parameter for each image, respectively. The most pronounced effect in differentiated PC12 cells expressing zRICH(H334A), when compared with neurons expressing RFP, was a 2.2 fold increase in the number of branching points per neuronal cell when compared with cells expressing RFP. The number of horizontal grid crosses increased by 1.3 fold, but it was not statistically significant. A modest, not statistically significant, 1.1 fold increase in neurite roots was observed. The bars show the average ± SEM; n = 20 random field images for both PC12-RFP and PC12-zRICH(H334A) differentiation assays. Statistics: * t-test, p < 0.05. The experiment presented is representative of 3 independent assays

The analysis of selected cells with the NeuronJ tracing procedure described above offers several advantages over our previous studies; however, this method still requires a lengthy procedure to find the cells that meet several criteria simultaneously (such as response to NGF, expression of high levels of the fluorescent proteins and separation from interfering cells), and to collect phase contrast and corresponding fluorescence microscopy images. Another time-consuming step is the computer-assisted tracing method. A second procedure was designed that avoids both the selection and tracing steps and analyzes images from random microscope fields by counting discrete parameters on phase contrast images, resulting in relatively rapid and efficient quantitations, albeit with the limitation of using all neurons observed rather than selecting those with high levels of expression. A summary diagram of the simplified procedure is presented in Fig. 3A, with an example of an image and the overlay quantitation of various parameters exhibited in Fig. 3B. The results of the analysis of a representative differentiation experiment analyzed by this procedure is presented in Fig. 3C. The images with the PC12-RFP-zRICH(H334A) stably transfected cells showed a statistically significant increase in the number of branching points per neuron when compared with PC12-RFP cells (approximately 2.2 fold in the representative experiment shown in Fig. 3C; 2.4 fold average increase was observed in 3 independent experiments). The differences in neurite roots per neuron were small and not statistically significant (1.1 fold in the experiment presented, 1.3 fold average for 3 experiments). Similarly, a modest increase in crosses with the superimposed horizontal grid lines was observed, probably as a consequence of the additional branches (1.3 fold increase for the experiment presented, but not statistically significant; 1.6 fold average for 3 independent experiments). The results obtained are in support of those observed with tracing methods, indicating that this procedure can detect the effect of zRICH on neuritogenesis, and suggesting that the average levels of expression of the protein are sufficient to cause a significant effect on branching. No significant differences on the percentages of cells responding to NGF were detected. These observations match previously published results that indicated that RICH proteins do not play a significant role in the initial response to NGF [45, 46].

### zRICH(H334A) expression promotes structural plasticity during neurite regeneration in PC12 cells

**Fig. 4:**
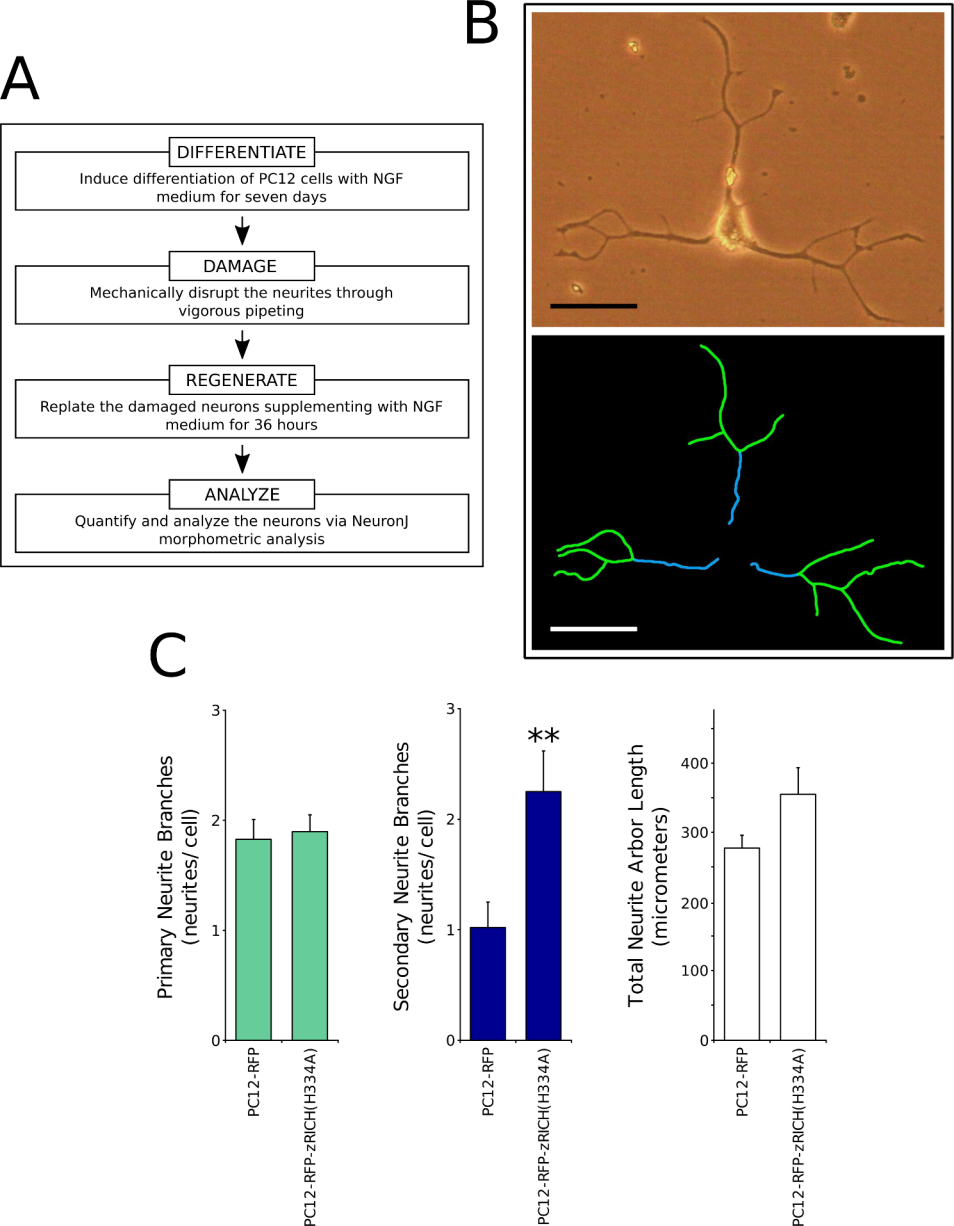
RFP-zRICH(H334A) expression promotes branching during neurite regeneration in PC12 cells. **A**: Summary of the neurite regeneration assay. The diagram summarizes the procedure to analyze the regrowth of neurites. Differentiated PC12 cells were subjected to mechanical damage and then allowed to regenerate neurites for a limited time in medium with NGF. Morphometric analysis was performed by the NeuronJ tracing procedure. **B**: Example of NeuronJ tracing of a PC12-RFP-zRICH(H334A) cell with an extensive regenerated neurite arbor after 36 h. The top image shows the phase contrast image and the bottom image shows NeuronJ neurite segment tracings (primary in blue and secondary in green). Scale bar represents 50 μm. **C**: Results of morphometric analysis of neurite regrowth assays with the stable transfectants. From left to right, the graphs represent the number of primary neurite branches, the number of secondary neurite segments, and the total neurite arbor length per cell, respectively. The most pronounced effect of the expression of zRICH(H334A) during neurite regeneration in PC12 cells (by comparison with cells expressing RFP) was again a significant increase of approximately 2.2 fold in secondary neurite segments. A smaller increase of approximately 1.3 fold was observed in the total neurite arbor length, but it was not statistically significant. The bars show the average ± SEM; n = 46 PC12-RFP and 39 PC12-zRICH(H334A) cells. Statistics: ** t-test, p < 0.01. The experiment presented is representative of 3 independent assays

After completion of a differentiation procedure, stable transfectant PC12 cells were subjected to mechanical stress, with the purpose of damaging the neurites of neurons. The cells were then allowed a short period of neurite regrowth to assess mainly regenerative processes [47], which were analyzed using the previously described NeuronJ tracing method. The steps of the procedure are summarized in Fig. 4A. Figure 4B shows an example of a PC12-zRICH(H334A) cell that exhibited extensive regrowth and neurite branching and the corresponding NeuronJ tracings. The expression of zRICH(H334A) in PC12 cells resulted in a significant increase in branching during neurite regrowth (approximately 2.2 fold increase of secondary neurite branches versus control cells, both for the experiment presented and for the average of 3 independent experiments), suggesting a similar effect of the protein on structural plasticity during neurite generation and regeneration. Also similar to the results for differentiation, a moderate increase in the total arbor length was observed, but was not statistically significant in individual experiments (approximately 1.3 fold average increase for the experiment presented and for the average of 3 independent experiments). This increase is again likely due mainly to an increase in secondary neurite branches (which are relatively short in length after 36 h of regeneration).

## Discussion

Teleost fish RICH proteins are neuronal GAPs that are induced in retinal ganglion cells during optic nerve regeneration [44]. They are transported down the growing axon and they accumulate at varicosities and membrane blebs often associated with formation of branches [48]. They are homologous with mammalian CNPases [49, 50], sharing significant sequence homology on the C-terminal two-thirds region [44], and they display several functional properties in common, such as phosphodiesterase activity (2’,3’-cyclic nucleotide 3’-phosphodiesterase) [42, 44], membrane association (through prenylated C-terminus) [45], interaction with tubulin, and the ability to promote the branching of membranous extensions [46]. Interestingly, the genes encoding these proteins show significant differences in regulation of expression in the CNS. Mammalian CNPases are abundantly expressed in the mammalian brain and spinal cord, mainly due to very high levels of expression in glial cells involved in myelination (oligodendrocytes). Conversely, RICH proteins demonstrated moderate levels of expression in the teleost brain and retina; however, in the retina, the protein was strongly induced in the neurons that are in the process of axon regeneration [44]. The process of nerve regeneration is very complex, its success depending on a variety of both extrinsic factors and intrinsic neuronal GAPs that modulate the regeneration capabilities of the tissue [12, 13, 17–19]. Due to the difference in the regulation of expression between teleost RICH proteins and mammalian CNPases, RICH proteins could be an important contributing factor for the higher intrinsic nerve regeneration capacity of neurons in the CNS of cold-blooded vertebrates. These proteins would not be available to the mammalian neuronal counterparts after neurite damage, giving interest to studies aiming to understand the roles of RICH proteins during neurite regrowth.

The PC12 cell line has been utilized extensively to study the process of neuritogenesis at both the cellular and molecular levels. Several neuronal GAPs have been demonstrated to increase the structural plasticity of PC12 cells, such as GAP43, CAP23, c-Jun, ATF3, etc., with expression of the proteins enhancing neuritogenesis in response to NGF or by combination with other factors that trigger differentiation [51–53]. The expression of zRICH in stable transfectant PC12 cells did not trigger generation of neurites by itself, but increased structural plasticity in response to NGF, although the effect occurred at later stages, by specifically promoting neurite branching [46]. Interestingly, a mutant version of the protein devoid of phosphodiesterase activity, termed zRICH(H334A), with a single amino acid substitution of a histidine located in the catalytic site, resulted in increased potency for enhancing neuritogenesis, suggesting a possible mechanism of self regulation and that this version of the protein could be used as a tool to enhance the intrinsic capacity of neurons for axon regeneration. These studies were limited by the need of fixing the cells and detecting expression through immunocytochemistry. To study the effects of this protein on neuritogenesis and nerve regeneration in further detail, new experimental procedures were designed that avoid the need for immunodetection of expression and facilitate the morphometric analysis of the differentiated cells. To be able to detect the expression levels of the zRICH(H334A) protein in living cells during the differentiation procedure, a eukaryotic plasmid was generated that encodes a fusion protein consisting of a monomeric version of RFP (DsRed-monomer) fused to the N-terminus of zRICH(H334A) (Fig. 1A). This design avoids interference with the C-terminal membrane localization motif. Stable transfectant PC12 cells were generated and demonstrated constitutive expression of the fusion protein by WB (Fig. 1B). Importantly, the protein allowed dynamic observation of expression under fluorescence microscopy and the capture of matching phase contrast and fluorescence microscopy images to facilitate the analysis of neuritogenesis (Fig. 2).

To study the effects of the expression of the fusion protein on neuritogenesis, a collection of images of differentiated neurons expressing zRICH were analyzed in detail by computer-assisted neurite tracing with the NeuronJ plugin of ImageJ image analysis software (Fig. 2B, C). The main effect of expression of RFP-zRICH(H334A) in PC12 cells during NGF induced neuritogenesis was an increase in neurite branching (approximately 2.4 fold increase versus control cells for the experiment shown in Fig. 2D). This effect matches well with previous results obtained with cells expressing unfused zRICH(H334A) [46], suggesting that the fusion of the fluorescent protein to the N-terminus did not block its function on promoting structural plasticity (although the magnitude of the effect detected was partially reduced). The RFP-zRICH(H334A) fusion protein was transported into the neurites and accumulated at branch points and terminals during differentiation, as shown in Fig. 2A and Supplementary Material 1: Fig. S1, matching with the pattern previously observed with the endogenous protein in neurons [48]. While the NeuronJ computer-assisted procedure facilitated the detailed analysis of effects on neuritogenesis, it still involves time-consuming tracing of neurites [54]. A second morphometric procedure was developed based on previous studies demonstrating successful neuronal differentiation analysis by applying stereological methods to counting frames [55, 56]. In contrast to the NeuronJ method, this second procedure bypassed the need for neurite tracing, and was applied to random field images collected following a normalized and systematic procedure to avoid overlap and to represent the global neuronal population in the culture dish. Importantly, this method avoids time-consuming scanning for isolated neurons (Fig. 3A). ImageJ software was then used to count certain parameters on each image (Fig. 3B). Interestingly, the random field procedure was also able to detect the effect of RFP-zRICH(H334A) on neurite branching (detected as an increase in the Branching Points per Neuronal Cell Count parameter ratio, Fig. 3C). For a comparison of several characteristics of these methods, please see Supplementary Material 2: Fig. S2.

Both the NeuronJ and random field procedures were able to detect changes in neurite morphology. While the random field procedure is more time-efficient, the differences in the two procedures could prove advantageous for specific experimental purposes, and the two methods can provide complementary information. For example, by comparing the results obtained with both methods, it is possible to discuss the effects of the protein at various levels of expression. The NeuronJ-based procedure can be applied to cells with specific levels of expression. For the experiments presented, cells with relatively high expression were analyzed (further subdivision was however not possible statistically as the number of cells matching requirements was limited). By utilizing pools of stable transfectants, the experiments avoided specific differences in the individual PC12 cells unrelated to the transfected gene. However, the pooled stable transfectants show characteristically wide variation of levels of expression for the introduced gene, which can span at least three orders of magnitude by flow cytometer analysis [57, 58]. Based on WB analyses, the average levels of protein expression in the PC12 stable transfectant pool exceed 0.5 ng of zRICH protein per µg of total cell protein (Fig. 1B), over 0.05% weight ratio, levels that would make it as abundant as some cytoskeleton-associated proteins [59, 60]. It can be speculated, based on estimates from the images obtained by fluorescence microscopy, that the NeuronJ analysis was performed with the neurons in the top 10% of levels of expression, possibly with levels higher than 10 fold above the average levels of the entire population. This would correspond to over 0.5% of the cellular protein weight ratio, becoming one of the most abundant proteins in the cell, probably close to the levels of tubulin itself. It is interesting to speculate, based on observations in previous WB analyses, that the levels of RICH expression in the stable transfectants could be similar to those in retinal ganglion cells in the process of nerve regeneration in zebrafish or goldfish (approximately 0.5-1 ng of RICH protein per µg of retina protein, where RGC are estimated to represent 5–10% of the retinal cells) [44]. The observed high levels of RICH expression during optic nerve regeneration, as well as previous functional analyses with RICH proteins and mammalian CNPases, suggested a role for these proteins in the regulation of the tubulin cytoskeleton and its interaction with the plasma membrane [45, 61]. The effects on neurite branching observed in the studies with PC12 cells expressing high levels of the RFP-zRICH(H334A) protein (Fig. 2D) are consistent with this hypothesis. On the other hand, the analysis of random fields allows an objective and relatively rapid analysis of a cell population by quantitating differentiation parameters on a per image basis. The results suggest that the effects on neurite branching observed with average levels of expression (Fig. 3C) match well with those observed with NeuronJ on cells with the highest levels of expression. Statistical comparisons of both variances and coefficients of variation for the effects detected with the two methods did not detect significant differences (F-tests, p > 0.05). Although not statistically significant, the classical analysis of selected cells with NeuronJ showed lower variation in the detection of the fold-effects on the parameters estimated (primary neurites, branching, arbor length). The procedure of analyzing random field images was used on a more diverse population of cells, probably explaining lower sensitivity to more moderate effects on parameters (such as for the Horizontal Grid Crosses, a parameter used to estimate neurite arbor length). Comparing effects of proteins at different levels on nerve regeneration can be important, to learn whether the effects do require very high levels of expression, or even if they differ at diverse levels. In several cases, for signaling molecules that are found at low levels in cells, artificial effects have been observed in experiments achieving high overexpression levels [62–64]. However, in this particular case, RICH and mammalian CNPases are expressed at fairly high levels in physiological systems, and the effects observed on the transfected PC12 neurons with the highest levels support a possible structural role on the tubulin cytoskeleton function during neuritogenesis. The experiments presented cannot tell whether the effects of the protein occur early during the generation of the branches from growth cones, or later by stabilization of the growing branches. Future studies analyzing video from continuous live microscopy could provide clues to answer this question.

Neurons obtained by differentiation of PC12 cells have been used previously to study neurite regrowth and regeneration [47, 65, 66]. When PC12 cells are differentiated with NGF, they become primed for rapid neurite regeneration through translational regulation and independent of new transcription [47]. Since the transfected cells can be analyzed for expression dynamically, without the need for immunodetection, they were used for neurite regeneration assays, by extending differentiation experiments to add a neurite injury protocol followed by a relatively short recovery period (Fig. 4A). The protocol was therefore designed to focus on regeneration rather than de novo differentiation. The expression of zRICH(H334A) promoted structural plasticity during neurite regeneration in PC12 cells by promoting a significant increase in branching (Fig. 4C). Consistent with our previous observations, the differentiation and regeneration studies suggest that while NGF is required for priming the formation and regrowth of neurites, expression of zRICH(H334A) resulted in higher levels of neurite arborization. A very important future direction of this project would be to address whether expression of zRICH-H334 could enhance the neurite regeneration capacity of primary mammalian neurons or of neurons derived from neural stem cells (NSCs). Another intriguing possibility for further future experiments is to test whether this protein can also have an effect on other diseases of the nervous system, such as in neurodegenerative diseases.

While RICH proteins are not present in mammalian neurons, our studies have shown that they can interact with the tubulin cytoskeleton and promote neurite plasticity in mammalian neurons derived from PC12 cells ([46]; work presented in this manuscript). The mammalian homologous CNPase protein also interacts with tubulin and promotes membrane extensions, but in oligodendrocytes instead of neurons. Our research findings and previous examples of successful interspecies gene rescue or reintroduction suggest that RICH proteins provide an interesting opportunity for reinvigorating the intrinsic capacity for axon regeneration in adult CNS mammalian neurons [67–69]. Combinatorial approaches involving overexpression of zRICH and other growth-associated proteins (targeting different intrinsic pathways), in synergy with strategies to counteract extrinsic inhibitory signals could facilitate persistent axon regeneration in mammals, as has been shown with other GAPs and treatments that block extrinsic inhibitory cues [23, 36, 70–72]. Targeted genetic modifications to precondition NSCs prior to transplantation could lead to increases in survival and structural plasticity [73–79]. We envision that combining overexpression of zRICH with that of other GAPs (that work using different intrinsic pathways than zRICH) and/or strategies that block extrinsic inhibitory signals could lead to synergistic effects on promoting neuronal plasticity in adult mammalian CNS neurons.

## Conclusions

A fusion protein combining a red fluorescent protein with a catalytically inactive mutant of the zebrafish RICH protein was generated, facilitating detection of expression levels. For the analysis of differentiation and regeneration experiments using PC12 cells, two methods were applied and combined to determine the details of the protein’s effect on neuritogenesis.

The experiments presented showed that the zRICH(H334A) protein not only has an effect on neurite branching during differentiation, but also during neurite regeneration, as detected for the first time in this article, suggesting that this protein contributes to a higher intrinsic capacity for axon repair in neurons.

Since RICH proteins originate from teleost fish and are not present in mammalian neurons, they could become tools to promote the intrinsic capacity for axon regeneration of adult neurons in the mammalian CNS through gene therapy approaches, perhaps beyond the internal capabilities of these neurons.

## Methods

### Plasmids

For the construction of the pDsRedC1-zRICH(H334A) plasmid, which expresses a fusion protein in which the DsRed RFP protein is attached to the amino-terminus of the catalytically inactive mutant zRICH(H334A), a *Bam*H I to *Bgl* II fragment obtained from the intermediate construct pBKSdCA-zRICH(H334A) was subcloned into the *Bam*H I site of pDsRed-monomer-C1 plasmid (Clontech). Proper insert orientation was confirmed by restriction endonuclease analysis. The intermediate construct was generated by the initial deletion of the *Cla* I to *Apa* I portion of the multiple cloning site (MCS) of pBKS-zRICH-WT plasmid [45] by restriction endonuclease digestion, fill-in reaction with the Klenow fragment of DNA Polymerase I and religation (the deletion removes a *Xho* I restriction site from the MCS), and the subsequent replacement of a *Xho* I to *Hind* III fragment by the corresponding fragment from the pKKR2-zRICH-H334A plasmid [42]. This replacement brings into the intermediate construct the mutation in codon 334 of the open reading frame (ORF) that substitutes the H to A in the encoded protein, and a *Bgl* II site right before the *Hind* III site. A plasmid map of the final construct and the entire sequence encoding the fusion protein are presented in Supplementary Material 3 and Supplementary Material 4.

### PC12 stable transfections

PC12 cells (rat pheochromocytoma) were obtained from the American Type Culture Collection (ATCC CRL-1721) and were cultured as previously described [80]. Approximately 750,000 cells per well were plated in multiwell-12 culture dishes pre-coated with polyethylenimine and cultured in RPMI-1640 medium supplemented with 10% Horse Serum and 5% Fetal Bovine Serum. The eukaryotic expression plasmids pDsRed-monomer-C1 and pDsRedC1-zRICH(H334A) were used for transfection. Transfections were performed by lipofection with Lipofectamine-2000 reagent (Invitrogen) using protocols recommended by the manufacturer. Forty-eight hours after transfection, the growth medium was supplemented with 500 µg/ml G418 (Promega). Transfected cells that integrate the plasmids into their genome were selected for 14–21 days by culture in medium with G418 and then pooled and further expanded. Expression of the transgenes in the stable transfectants was analyzed by WB with anti-RICH polyclonal antibody or with anti-RFP antibody (Rockland) following protocols described previously [45]. For the protein analysis, cell lysates were separated by SDS-PAGE and blotted onto a nitrocellulose membrane (50 µg of total protein per cell lysate to allow sensitive detection of protein expression in the stable transfectants). Immunoblotting with anti-Tubulin antibody (Sigma) was performed as previously described [46]. Chemiluminescence signal was detected using a Kodak 440 Imager Station.

### PC12 differentiation assays and microscopy

PC12 stable transfectants were seeded at low densities in multiwell-12 culture dishes pre-coated with polyethylenimine (12,000 cells per well). Differentiation was induced by treatment with 100 ng/ml of NGF (Sigma) in RPMI-1640 medium with low serum content (1% Fetal Bovine Serum). The differentiation medium was replaced every 2 days. The cells were taken from the incubator and observed daily under both brightfield phase contrast and fluorescence microscopy (Olympus CK40), to monitor neurite growth and fluorescent protein expression. Microphotographs were obtained with a Pixera Penguin digital camera. Full image sets were obtained after 7 days of treatment with differentiation medium and utilized for detailed morphometric analyses.

### Computer-assisted morphometric analysis - NeuronJ tracing procedure

The plates were scanned under fluorescence microscopy to find cells matching several criteria appropriate for inclusion in the analysis: neuronal differentiation in response to NGF (criterion set as having either a neurite arbor of at least 4 body lengths, or at least one neurite longer than 3 body lengths), good levels of expression of protein of interest (detectable by fluorescence microscopy), and being well located (well separated from other cells to allow neurite assignments, as well as location in the central part of the dish well to allow good phase contrast imaging).

Neurite quantification was performed by employing ImageJ software [81] in conjunction with a plugin – NeuronJ, which aids in semiautomatic tracing of neurites [54]. For consistency with previous analyses [46], neurite segments were labeled either as primary – any neurite emanating from the cell body (of at least one cell body length), or secondary – any neurite segment of at least one cell body length not originating directly from the cell body (i.e., neurite branch segments that originate from another neurite). The restriction of counting only segments that are of at least one body length was applied to avoid counting small membrane extensions or growth cone filopodia. Please see the specific number of neurons analyzed for the experiments presented in the figure legends.

### Computer-assisted morphometric analysis - random field analysis procedure

Differentiated PC12 stable transfectant cells were observed under brightfield phase contrast microscopy to collect image sets of non-overlapping random fields of view. For procedure normalization, the following guidelines and criteria were utilized to obtain the images included in the analysis: images were obtained from the central area of the well that shows high quality phase contrast visualization, an alternating scanning pattern (left-to-right, down, right-to-left, down, etc.) was followed to capture images of non-overlapping fields, and fields with large clusters of tightly bound cells were skipped. The last selection criterion is required since PC12 cells have the tendency to form clusters, and relatively large clusters of strongly attached cells would reduce the quality of the analysis (assignment of several parameters is very difficult in these clusters, and response to NGF in the clusters is not normal as cells deep in the cluster have limited access to the neurotrophin).

The set of random field images (or a subset from the collection, chosen using a random number generator) was then analyzed utilizing the image processing software ImageJ. For each random field image, the plugins “Cell Counter” and “Grid” were used to assist the investigator with the numerical quantification of the following parameters: Total Cell Count, Neuronal Cell Count, Neurite Roots, Terminal Neurite Ends, Branching Points, and Horizontal Grid Crosses. The Total Cell Count was defined as the total number of cells within the field. The Neuronal Cell Count was defined as the number of differentiated neurons in the field (following the criterion described earlier: having either a neurite arbor of at least 4 body lengths, or at least one neurite longer than 3 body lengths). These two cell counts were used to determine the degree of differentiation for each assay. Four additional parameters, Neurite Roots, Terminal Neurite Ends, Branching Points and Horizontal Grid Crosses, were only quantified on neurites from identified differentiated neurons (or for neurites entering from areas surrounding the field of view). Neurite Roots were identified as the point on a neurite that stems directly from the cell body, and they were quantified to estimate the average number of primary neurites generated per differentiated neuron (by normalizing through division by the Neuronal Cell Count parameter for each image). Terminal Neurite Ends were identified as the ending of any neurite, which in combination with other parameters (Primary Neurite Roots and Branching Points) provide information about the overall level of arborization and number of secondary neurites per neuron. A Branching Point was identified as any point along a neurite where the neurite splits to form secondary neurites. A horizontal grid of lines was superimposed on the image using the ImageJ plugin “Grid” to identify Horizontal Grid Crosses, defined as any point along a neurite that crossed a grid line, and this parameter was used as an estimate of relative neurite arbor lengths by normalization for each image through division by its corresponding Neuronal Cell Count parameter.

### Neurite regeneration assays

A neurite regeneration assay was developed based on published procedures that use PC12 cells to assess neurite regrowth following mechanical damage [47, 65, 66]. After completing a 7-day differentiation “priming” procedure, performed as described above, the cells were mechanically detached by forceful pipetting with NGF-free medium, causing neurite injury on the differentiated neurons. The recovered cells were then replated on a new PEI coated multi-well-12 culture dish and cultured with low serum differentiation media (the replated cells, at time 0 h post-injury, were confirmed to have spherical shape and no neurites; please see Supplementary Material 5: Fig. S5). Neurite regrowth capabilities were studied 36 h post-injury by obtaining images of neuronal cells selected for analysis with the NeuronJ tracing procedure, as described above. The 36 h time point was chosen since it facilitated assessment of branching in regenerated neurites. The expression of the proteins was reduced moderately after neurite injury, probably due to the stress of the mechanical detachment procedure (please see Supplementary Material 6: Fig. S6).

### Statistical analyses and reproducibility

Two-sample unpaired student’s t-tests were used to evaluate the statistical significance of the quantified data, to compare the results obtained with cells expressing RFP-zRICH(H334A) protein versus control cells expressing RFP, estimating the probability (p) of obtaining the result observed assuming that the two samples came from the same population. The averages of the two samples were considered statistically different whenever p was lower than 0.05. All measured data are expressed as mean ± standard error of the mean (SEM). Variances and coefficients of variation for the effects detected with both methods were compared and evaluated for statistical significance through F-tests. The analyses were conducted with a Microsoft Excel worksheet designed for this purpose or using IBM SPSS 22 (IBM Corporation) software. The quantitation results were reproducible in independent experiments; the methods yielded similar results when image sets were quantitated by different scientists or by utilizing a blind protocol with coded images.

### Electronic supplementary material

Below is the link to the electronic supplementary material.

Supplementary Material 1: RFP-zRICH(H334A) localizes to the neurite branching points during PC12 differentiation.

Supplementary Material 2: Advantages and drawbacks of various morphometric analysis procedures for the analysis of differentiation assays with PC12 cells.

Supplementary Material 3: Schematic representation of the pDsRed-zRICH(H334A) plasmid.

Supplementary Material 4: DNA sequence from the pDsRed-zRICH(H334A) plasmid that encodes the RFP-zRICH(H334A) fusion protein.

Supplementary Material 5: Images of neurite regeneration assay at 0 hours after injury.

Supplementary Material 6: Comparison of RFP-zRICH(H334A) expression before and after mechanical injury in stable transfectant cells.

Supplementary Material 7: Original unprocessed images of full-length blots.

## Data Availability

The datasets used and/or analyzed during the current study are available from the corresponding author on reasonable request.

## List of Abbreviations

CNPase: 2’3’-cyclic nucleotide 3’-phosphodiesterase
GAP: Growth associated protein
NGF: Nerve growth factor
PAGE: Polyacrylamide gel electrophoresis
RAG: Regeneration associated gene
RFP: Red fluorescent protein
RGC: Retinal ganglion cell
RICH: Regeneration induced CNPase homolog
SDS: Sodium dodecyl sulfate
WB: Western blot
WT: Wild-type
