# Supplementary material for: Enhancing structural plasticity of PC12 neurons during differentiation and neurite regeneration with a catalytically inactive mutant version of the zRICH protein
Source: BMC Neurosci. 2023 Aug 23;24:43. doi: 10.1186/s12868-023-00808-1 (PMC10463786; doi:10.1186/s12868-023-00808-1)
Supplement: Supplementary file 1 — Supplementary Material 1: RFP-zRICH(H334A) localizes to the neurite branching points during PC12 differentiation. [file 12868_2023_808_MOESM1_ESM.pdf]

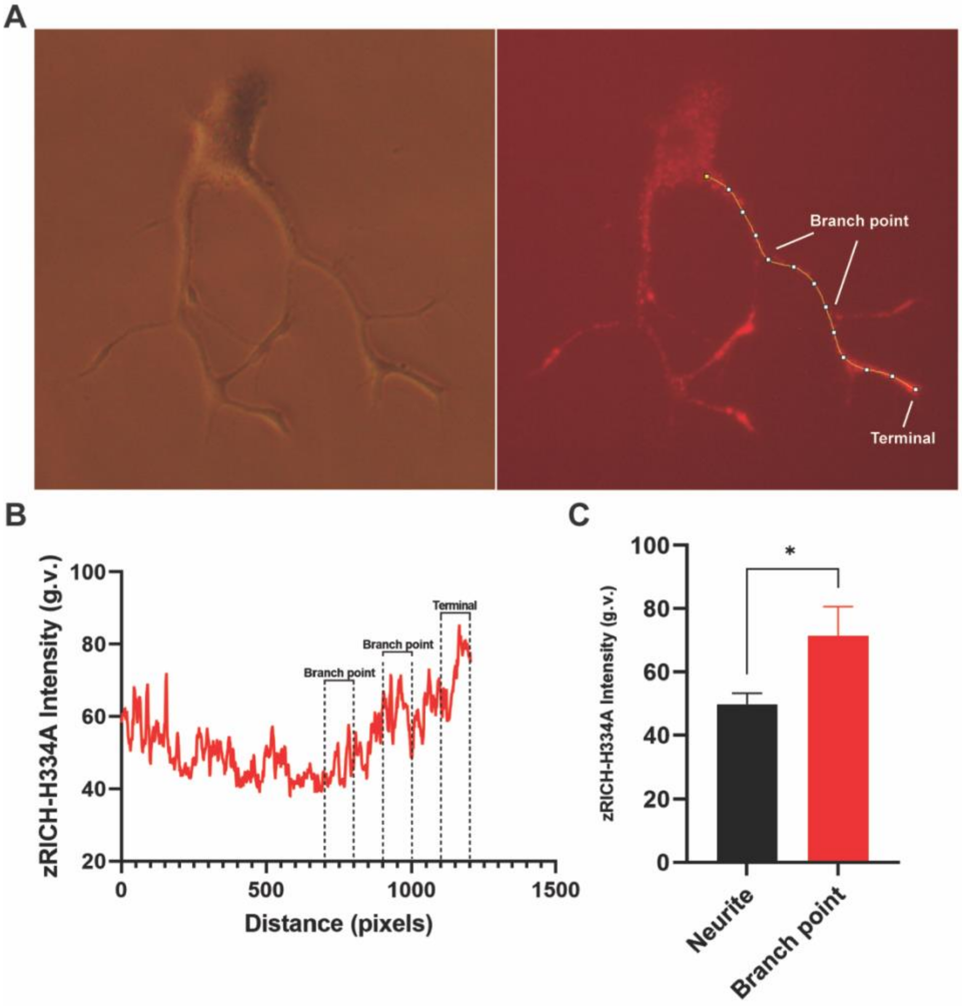

Supplementary Figure 1: RFP-zRICH(H334A) localizes to the neurite branching points during PC12 differentiation. **A:** Example of ImageJ tracing of a differentiated PC12-RFP-zRICH(H334A) cell using the segmented line tool. The left panel shows the phase contrast microscopy image, and the right panel shows the fluorescence image with the trace. **B:** Representative density profile plot of the grey values (g.v.) along the neurite trace. **C:** Graph representing RFP-zRICH(H334A) intensity (g.v.) in neurites vs. neurite branch points. The bars show the average ± SEM; n= 4 PC12-RFP-zRICH(H334A) cells. Statistics: * t-test, $p<0.05$.
