# Supplementary material for: Enhancing structural plasticity of PC12 neurons during differentiation and neurite regeneration with a catalytically inactive mutant version of the zRICH protein
Source: BMC Neurosci. 2023 Aug 23;24:43. doi: 10.1186/s12868-023-00808-1 (PMC10463786; doi:10.1186/s12868-023-00808-1)
Supplement: Supplementary file 2 — Supplementary Material 2: Advantages and drawbacks of various morphometric analysis procedures for the analysis of differentiation assays with PC12 cells. [file 12868_2023_808_MOESM2_ESM.pdf]

	Immunohistochemistry + Manual Morphometric Analysis[1]	NeuronJ Tracing[2]	Random Field Analysis[3]	Sholl Analysis[4]
**Advantages**	Allows selection by expression levels<br><br>Provides multiple morphometric parameters: neurite roots, branching points, neurite length (as absolute values for each neuron)	No sample fixation or immunohistochemistry required<br><br>Allows selection by expression levels (by fluorescence microscopy)<br><br>Provides multiple morphometric parameters: neurite roots, branching points, neurite length (as absolute values for each neuron)<br><br>Open source software available	No sample fixation or immunohistochemistry required<br><br>No requirement of scanning for isolated neurons (compatible with complex images with multiple neurons)<br><br>No requirement for neurite tracing<br><br>Provides multiple morphometric parameters: neurite roots, branching points, grid crosses[5] (as estimates per neuron)<br><br>Open source software available	No sample fixation or immunohistochemistry required<br><br>Allows selection by expression levels (by fluorescence microscopy)<br><br>No requirement for neurite tracing<br><br>Open source software available
**Drawbacks**	Requires fixation and immunohistochemistry<br><br>Requires scanning for isolated neurons<br><br>Requires neurite tracing (manual)	Requires scanning for isolated neurons<br><br>Requires neurite tracing (computer-assisted)	Does not allow selection by expression levels	Requires scanning for isolated neurons<br><br>Output is a single graph for each neuron or a representative profile averaging data for multiple neurons; not conducive to determine specific parameters such as neurite roots or branching points

Supplementary Figure 2. Advantages and drawbacks of various morphometric analysis procedures for the analysis of differentiation assays with PC12 cells, comparing the NeuronJ Tracing and Random Field Analysis procedures (utilized in this article) with the Immunohistochemistry + Manual Morphometric Analysis procedure utilized in our previous publication, and with the well-established Sholl method. [1] Pathi *et al.*, 2012, Brain Res., 1474, 29–39. [2] This article; based on Meijering *et al.*, 2004, Cytometry 58A, 167-176. [3] This article; based on Rønn *et al.*, 2000, J. Neurosci. Methods, 100, 25–32. [4] Sholl, 1953, J. Anat., 87, 387-406; Ferreira *et al.*, 2014, Nature Methods, 11, 982-984. [5] Grid crosses per neuron used as relative parameter for comparative analyses of neurite arbor lengths (estimate).
