# Supplementary material for: Enhancing structural plasticity of PC12 neurons during differentiation and neurite regeneration with a catalytically inactive mutant version of the zRICH protein
Source: BMC Neurosci. 2023 Aug 23;24:43. doi: 10.1186/s12868-023-00808-1 (PMC10463786; doi:10.1186/s12868-023-00808-1)
Supplement: Supplementary file 3 — Supplementary Material 3: Schematic representation of the pDsRed-zRICH(H334A) plasmid. [file 12868_2023_808_MOESM3_ESM.pdf]

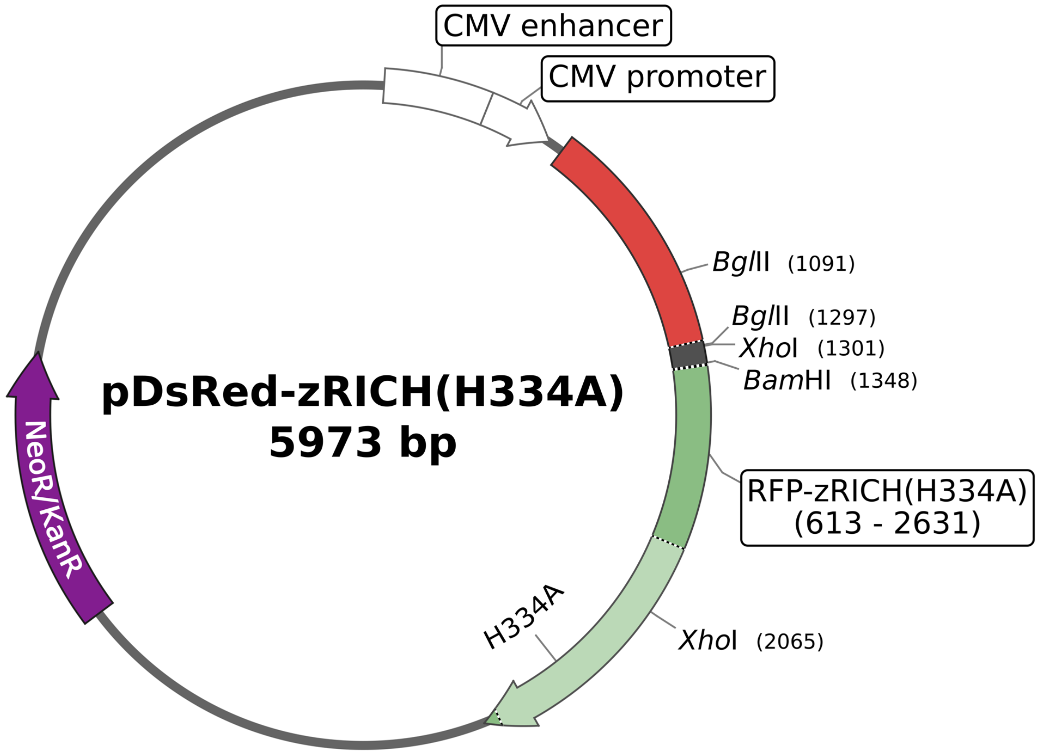

Supplementary Figure 3. Schematic representation of the pDsRed-zRICH(H334A) plasmid. The plasmid contains a CMV promoter that drives the expression of an open reading frame (613-2631) encoding the 672 amino acid long RFP-zRICH(H334A) fusion protein. For the encoded protein, the RFP component (248 amino acids) contains the DsRed-monomer (red), and is fused to the amino-terminus of zRICH(H334A) through a 23 amino acid long peptide (grey) derived from the plasmid polylinker region. The NeoR/KanR gene provides resistance against G418, a neomycin analog. The domains of the zRICH protein portion are indicated in darker and lighter shades of green: acidic domain (amino acids 249 - 419, dark green shade), CNPase homology domain (amino acids 420 - 658, light green shade), membrane localization domain (amino acids 659 - 672, dark green shade). The amino acid substitution from H to A at position 334 of the zRICH part of the protein is also indicated on the plasmid map.
