# Supplementary material for: Enhancing structural plasticity of PC12 neurons during differentiation and neurite regeneration with a catalytically inactive mutant version of the zRICH protein
Source: BMC Neurosci. 2023 Aug 23;24:43. doi: 10.1186/s12868-023-00808-1 (PMC10463786; doi:10.1186/s12868-023-00808-1)
Supplement: Supplementary file 4 — Supplementary Material 4: DNA sequence from the pDsRed-zRICH(H334A) plasmid that encodes the RFP-zRICH(H334A) fusion protein. [file 12868_2023_808_MOESM4_ESM.pdf]

```
                    20                  40                  60                  80
          *         *         *         *         *         *         *         *         *
  1 cgttacataacttacggtaaatggcccgcctggctgaccgcccaacgacccccgcccattgacgtcaataatgacgtatgttcccatagt 90
```

CMV enhancer and promoter

```
          100                 120                 140                 160                 180
          *         *         *         *         *         *         *         *         *
 91 aacgccaatagggactttccattgacgtcaatgggtggagtatttacggtaaactgcccacttggcagtacatcaagtgtatcatatgcc 180

                    200                 220                 240                 260
          *         *         *         *         *         *         *         *         *
181 aagtacgccccctattgacgtcaatgacggtaaatggcccgcctggcattatgcccagtacatgaccttatgggactttcctacttggca 270

          280                 300                 320                 340                 360
          *         *         *         *         *         *         *         *         *
271 gtacatctacgtattagtcatcgctattaccatggtgatgcggttttggcagtacatcaatgggcgtggatagcggtttgactcacgggg 360

                    380                 400                 420                 440
          *         *         *         *         *         *         *         *         *
361 atttccaagtctccaccccattgacgtcaatgggagtttgttttggcaccaaaatcaacgggactttccaaaatgtcgtaacaactccgc 450

          460                 480                 500                 520                 540
          *         *         *         *         *         *         *         *         *
451 cccattgacgcaaatgggcggtaggcgtgtacggtgggaggtctatataagcagagctggtttagtgaaccgtcagatccgctagcgcta 540

                    560                 580                 600                 620
          *         *         *         *         *         *         *         *         *
541 ccggtcgccaccATGGACAACACCGAGGACGTCATCAAGGAGTTCATGCAGTTCAAGGTGCGCATGGAGGGCTCCGTGAACGGCCACTAC 630
                M  D  N  T  E  D  V  I  K  E  F  M  Q  F  K  V  R  M  E  G  S  V  N  G  H  Y   26
```

RFP

```
          640                 660                 680                 700                 720
          *         *         *         *         *         *         *         *         *
631 TTCGAGATCGAGGGCGAGGGCGAGGGCAAGCCCTACGAGGGCACCCAGACCGCCAAGCTGCAGGTGACCAAGGGCGGCCCCCTGCCCTTC 720
    F  E  I  E  G  E  G  E  G  K  P  Y  E  G  T  Q  T  A  K  L  Q  V  T  K  G  G  P  L  P  F   56

                    740                 760                 780                 800
          *         *         *         *         *         *         *         *         *
721 GCCTGGGACATCCTGTCCCCCCAGTTCCAGTACGGCTCCAAGGCCTACGTGAAGCACCCCGCCGACATCCCCGACTACATGAAGCTGTCC 810
    A  W  D  I  L  S  P  Q  F  Q  Y  G  S  K  A  Y  V  K  H  P  A  D  I  P  D  Y  M  K  L  S   86

          820                 840                 860                 880                 900
          *         *         *         *         *         *         *         *         *
811 TTCCCCGAGGGCTTCACCTGGGAGCGCTCCATGAACTTCGAGGACGGCGGCGTGGTGGAGGTGCAGCAGGACTCCTCCCTGCAGGACGGC 900
    F  P  E  G  F  T  W  E  R  S  M  N  F  E  D  G  G  V  V  E  V  Q  Q  D  S  S  L  Q  D  G   116
```

```
                       920                 940                 960                 980
              *         *         *         *         *         *         *         *         *
 901 ACCTTCATCTACAAGGTGAAGTTCAAGGGCGTGAACTTCCCCGCCGACGGCCCCGTAATGCAGAAGAAGACTGCCGGCTGGGAGCCCTCC 990
     T  F  I  Y  K  V  K  F  K  G  V  N  F  P  A  D  G  P  V  M  Q  K  K  T  A  G  W  E  P  S   146

            1000                1020                1040                1060                1080
              *         *         *         *         *         *         *         *         *
 991 ACCGAGAAGCTGTACCCCCAGGACGGCGTGCTGAAGGGCGAGATCTCCCACGCCCTGAAGCTGAAGGACGGCGGCCACTACACCTGCGAC 1080
     T  E  K  L  Y  P  Q  D  G  V  L  K  G  E  I  S  H  A  L  K  L  K  D  G  G  H  Y  T  C  D   176

                      1100                1120                1140                1160
              *         *         *         *         *         *         *         *         *
1081 TTCAAGACCGTGTACAAGGCCAAGAAGCCCGTGCAGCTGCCCGGCAACCACTACGTGGACTCCAAGCTGGACATCACCAACCACAACGAG 1170
     F  K  T  V  Y  K  A  K  K  P  V  Q  L  P  G  N  H  Y  V  D  S  K  L  D  I  T  N  H  N  E   206

            1180                1200                1220                1240                1260
              *         *         *         *         *         *         *         *         *
1171 GACTACACCGTGGTGGAGCAGTACGAGCACGCCGAGGCCCGCCACTCCGGCTCCCAGTCCGGACTCAGATCTCGAGCTCAAGCTTCGAAT 1260
     D  Y  T  V  V  E  Q  Y  E  H  A  E  A  R  H  S  G  S  Q  S  G  L  R  S  R  A  Q  A  S  N   236
                                                              Polylinker

                      1280                1300                1320                1340
              *         *         *         *         *         *         *         *         *
1261 TCTGCAGTCGACGGTACCGCGGGCCCGGGATCCACCATGGAAGCTGAACAGAATCAGGAAGTTCAAGAGGCAGTTCCTGAGACGCAGGAA 1350
     S  A  V  D  G  T  A  G  P  G  S  T  M  E  A  E  Q  N  Q  E  V  Q  E  A  V  P  E  T  Q  E   266
                                         zRICH(H334A)

            1360                1380                1400                1420                1440
              *         *         *         *         *         *         *         *         *
1351 GTGGCGGCTCAGCAAGAGGAGAAATCAGAGCCCAAGTCCGAAGAGGCGCCTCAGGCTCCTTCAGAAGCAGCAGCAGATCCTCCAGCAGCG 1440
     V  A  A  Q  Q  E  E  K  S  E  P  K  S  E  E  A  P  Q  A  P  S  E  A  A  A  D  P  P  A  A   296

                      1460                1480                1500                1520
              *         *         *         *         *         *         *         *         *
1441 GCCCCTGAACCGGAGAAGCCCCAAGAGACAGAGCCTTCTGCCGAGGAACAGCAGAAAGCAACAGAATCTGCAGCATCTCCTGCCAAACCC 1530
     A  P  E  P  E  K  P  Q  E  T  E  P  S  A  E  E  Q  Q  K  A  T  E  S  A  A  S  P  A  K  P   326

            1540                1560                1580                1600                1620
              *         *         *         *         *         *         *         *         *
1531 TCTGAACCCGAGGCAAAATCTCCAGAGGATTCCTCTGAAAAAACCCCTGAGCAGCAGCAGAAGTCGTCCGAGGAGCCATCGCTTCAAGTC 1620
     S  E  P  E  A  K  S  P  E  D  S  S  E  K  T  P  E  Q  Q  Q  K  S  S  E  E  P  S  L  Q  V   356

                      1640                1660                1680                1700
              *         *         *         *         *         *         *         *         *
1621 AATTCTGAGCCCGAGAAGCAGGAGGAGGAAGCTGTGAAAGAGGCTGAGTCTAAGAAAGAGGAGCCTCTCAAAGAGGCGGAGTCCAAACCT 1710
     N  S  E  P  E  K  Q  E  E  E  A  V  K  E  A  E  S  K  K  E  E  P  L  K  E  A  E  S  K  P   386
     z

            1720                1740                1760                1780                1800
              *         *         *         *         *         *         *         *         *
1711 GCTGCTGTGAATGAAGTCAAGCCAGAGGAGTCTGAGAAAAGTGAGACGACAAAAGCAGAAGGAGAAAAGGTACAAGTGCCCGAGGCTGAT 1800
     A  A  V  N  E  V  K  P  E  E  S  E  K  S  E  T  T  K  A  E  G  E  K  V  Q  V  P  E  A  D   416
```

```
                1820              1840              1860              1880
        *         *         *         *         *         *         *         *         *
1801 GGAGTTCAAGCTGAGCCTCCAAAAGAGACCGAACCTGAGGAGAAGAAACCAGAGCTGCCACTCTTTTATGGCTGGTTCCTGCTTAAAGAG 1890
     G  V  Q  A  E  P  P  K  E  T  E  P  E  E  K  K  P  E  L  P  L  F  Y  G  W  F  L  L  K  E   446

      1900              1920              1940              1960              1980
        *         *         *         *         *         *         *         *         *
1891 GAAGAGGAGCGAATTAAGTGTGCAACCATGGACTTCCTAAAGACGCTGGATACATTGGAAGCCTTCAAAGAACACATAAGTGAATTTACG 1980
     E  E  E  R  I  K  C  A  T  M  D  F  L  K  T  L  D  T  L  E  A  F  K  E  H  I  S  E  F  T   476

                2000              2020              2040              2060
        *         *         *         *         *         *         *         *         *
1981 AGTGAGGCAAATAAAGAGGTGGATCTCGAGCAGTATTTCCAGAACCCACTGCCACTCCACTGCACTACAAAGTTCTGTGACTACGGCAAA 2070
     S  E  A  N  K  E  V  D  L  E  Q  Y  F  Q  N  P  L  P  L  H  C  T  T  K  F  C  D  Y  G  K   506

      2080              2100              2120              2140              2160
        *         *         *         *         *         *         *         *         *
2071 GCAGAAGGAGCAAAAGAGTACGCAGAGATGCAGGTGGTCAAGGAAGCGACTGGCAAATCAGAGGAGCTCTCGGTTACCGCTCTCATCGTG 2160
     A  E  G  A  K  E  Y  A  E  M  Q  V  V  K  E  A  T  G  K  S  E  E  L  S  V  T  A  L  I  V   536

                2180              2200              2220              2240
        *         *         *         *         *         *         *         *         *
2161 ACCCCTCGTACATTCGGGGCACGTGTGGCTTTGACCGAAACCCAGCTGAATCTGTGGCCTGAGGGAGAGGATAAAGTTGGGGTTGCTCCA 2250
     T  P  R  T  F  G  A  R  V  A  L  T  E  T  Q  L  N  L  W  P  E  G  E  D  K  V  G  V  A  P   566

      2260              2280              2300              2320              2340
        *         *         *         *         *         *         *         *         *
2251 ACCCTCTTGCCCGGCGTAGAGTCTCTGCCGGCTGGCAGTCGCGCCGCCGTCACATTAGGCTGCTCGGCTGGTGTGGAGGCGGTTCAGACA 2340
     T  L  L  P  G  V  E  S  L  P  A  G  S  R  A  A  V  T  L  G  C  S  A  G  V  E  A  V  Q  T   596
                                               H334A

                2360              2380              2400              2420
        *         *         *         *         *         *         *         *         *
2341 GGTCTGGATCTGCTGGAGATCCTGGTTCTGCAGAAGGAGGGTAAGGAGGGCACTCAGGTGGAGATGGATATGGGCACTTTGTCCTACCTT 2430
     G  L  D  L  L  E  I  L  V  L  Q  K  E  G  K  E  G  T  Q  V  E  M  D  M  G  T  L  S  Y  L   626

      2440              2460              2480              2500              2520
        *         *         *         *         *         *         *         *         *
2431 AGCGAGGGCCGCTGGTACCTAGCCCTGAGGGAAGCCATTACCGCAGACACCACCTTCTCTAGCTTCTCTGAAGACAAGCCTGTCAGCGAC 2520
     S  E  G  R  W  Y  L  A  L  R  E  A  I  T  A  D  T  T  F  S  S  F  S  E  D  K  P  V  S  D   656

                2540              2560
        *         *         *         *         *
2521 CAGGGCAAAAAGGATGGAGAGAAGAAAAAGAAAAAGTGTACCATTCTGTGA 2571
     Q  G  K  K  D  G  E  K  K  K  K  K  C  T  I  L  *   672
```

Supplementary Figure 4. DNA sequence from the pDsRed-zRICH(H334A) plasmid that encodes the RFP-zRICH(H334A) fusion protein. The sequence presented encompasses the CMV promoter region and the entire ORF for the 672 amino acid long RFP-zRICH(H334A) fusion protein, with the one-letter amino acid code shown below. The 225 amino acid long DsRed-monomer RFP (red) is fused to the amino-terminus of the 424 amino acid long zRICH(H334A) via a plasmid-derived 23 amino acid long peptide (grey). The domains of the zRICH protein portion are indicated in darker and lighter shades of green: acidic domain (amino acids 249 - 419, dark green shade), CNPase homology domain (amino acids 420 - 658, light green shade), membrane localization domain (amino acids 659 - 672, dark green shade). The amino acid substitution from H to A at position 334 of the zRICH part of the protein is also indicated in the sequence.
