# Supplementary material for: Enhancing structural plasticity of PC12 neurons during differentiation and neurite regeneration with a catalytically inactive mutant version of the zRICH protein
Source: BMC Neurosci. 2023 Aug 23;24:43. doi: 10.1186/s12868-023-00808-1 (PMC10463786; doi:10.1186/s12868-023-00808-1)
Supplement: Supplementary file 5 — Supplementary Material 5: Images of neurite regeneration assay at 0 hours after injury. [file 12868_2023_808_MOESM5_ESM.pdf]

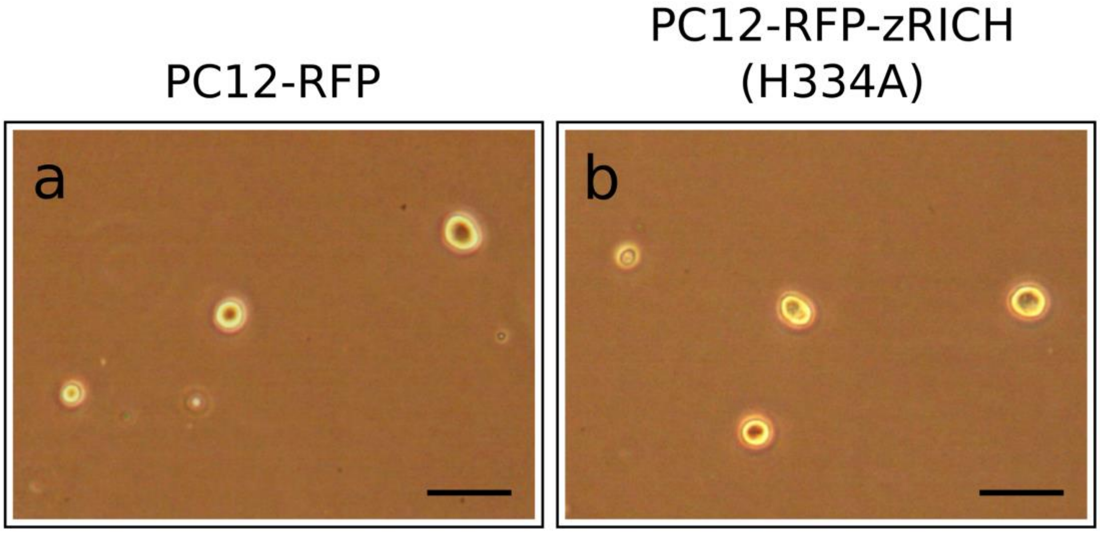


**Supplementary Figure 5.** Images of neurite regeneration assay at 0 hours after injury. Differentiated PC12 stable transfectant cells were subjected to mechanical damage and then re-plated in low serum differentiation medium. Phase contrast microscopy images show PC12-RFP (panel a) and PC12-RFP-zRICH(H334A) stable transfectant cells (panel b) re-plated just after the forceful pipetting procedure (0 h post-injury). Microphotographs show that these cells have lost their neurites, exhibiting a spherical shape. Scale bars represent 50 μm.
