# Supplementary material for: Enhancing structural plasticity of PC12 neurons during differentiation and neurite regeneration with a catalytically inactive mutant version of the zRICH protein
Source: BMC Neurosci. 2023 Aug 23;24:43. doi: 10.1186/s12868-023-00808-1 (PMC10463786; doi:10.1186/s12868-023-00808-1)
Supplement: Supplementary file 6 — Supplementary Material 6: Comparison of RFP-zRICH(H334A) expression before and after mechanical injury in stable transfectant cells. [file 12868_2023_808_MOESM6_ESM.pdf]

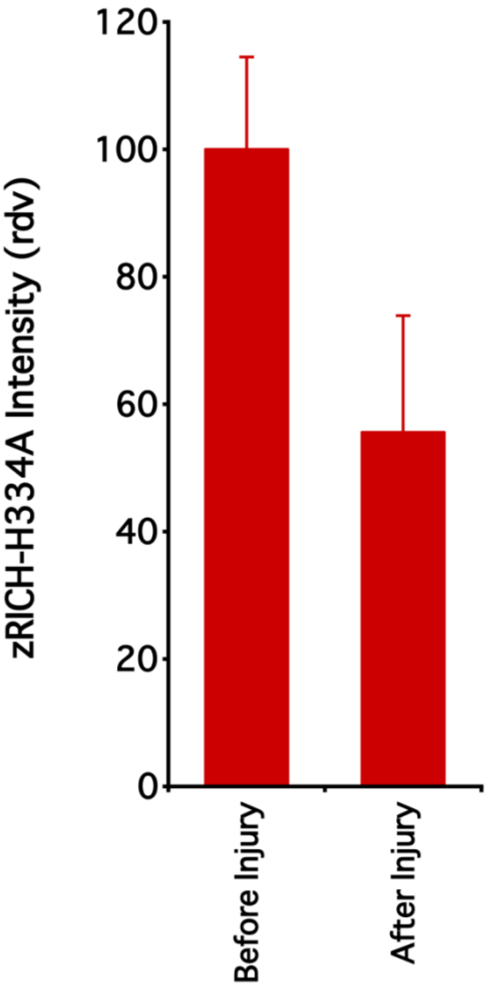

Supplementary Figure 6: Comparison of RFP-zRICH(H334A) expression before and after mechanical injury in stable transfectant cells. The graph shows the relative density levels (rdl) of expression 36 hours after the forced mechanical detachment procedure compared to pre-injury levels. The moderate reduction in expression (56% of pre-injury levels) may be due to the cellular stress of mechanical injury, as a similar reduction was observed in PC12-RFP cells after injury (43% of pre-injury levels). Bars represent mean ± SEM; n= 15 PC12-RFP-zRICH(H334A) cells. However, the reduction in RFP-zRICH(H334A) expression was not found to be statistically significant (t-test, p = 0.068).
