# Supplementary material for: Enhancing structural plasticity of PC12 neurons during differentiation and neurite regeneration with a catalytically inactive mutant version of the zRICH protein
Source: BMC Neurosci. 2023 Aug 23;24:43. doi: 10.1186/s12868-023-00808-1 (PMC10463786; doi:10.1186/s12868-023-00808-1)
Supplement: Supplementary file 7 — Supplementary Material 7: Original unprocessed images of full-length blots. [file 12868_2023_808_MOESM7_ESM.pdf]

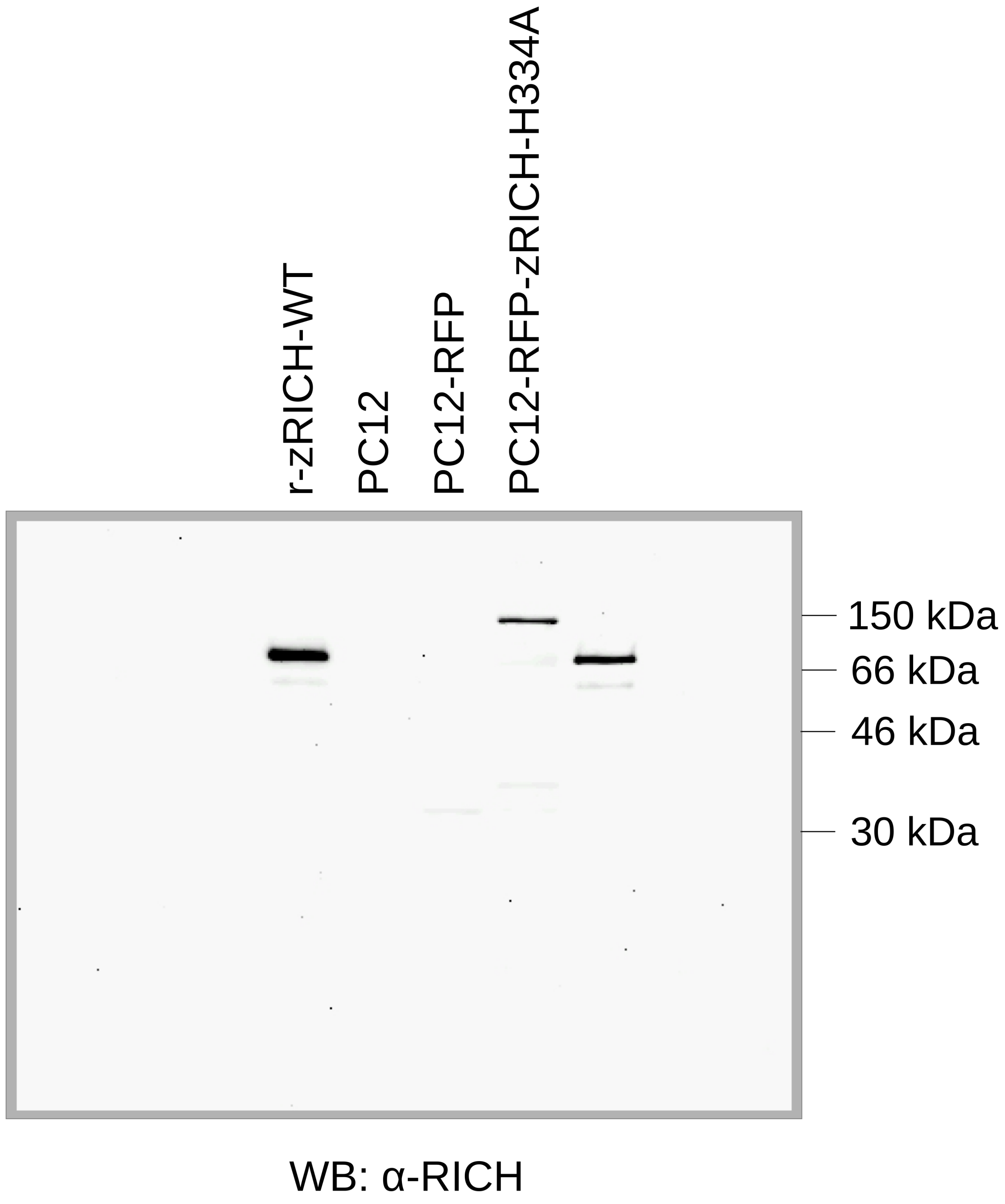

Western blot - Membrane 1 - anti-RICH. The figure was prepared with the original unprocessed (uncropped) image obtained from the Kodak 440 Imager Station. A box filled with grey color was used as background to facilitate the identification of the edges of the image. Please see Figure 1 for comparison and detailed description.

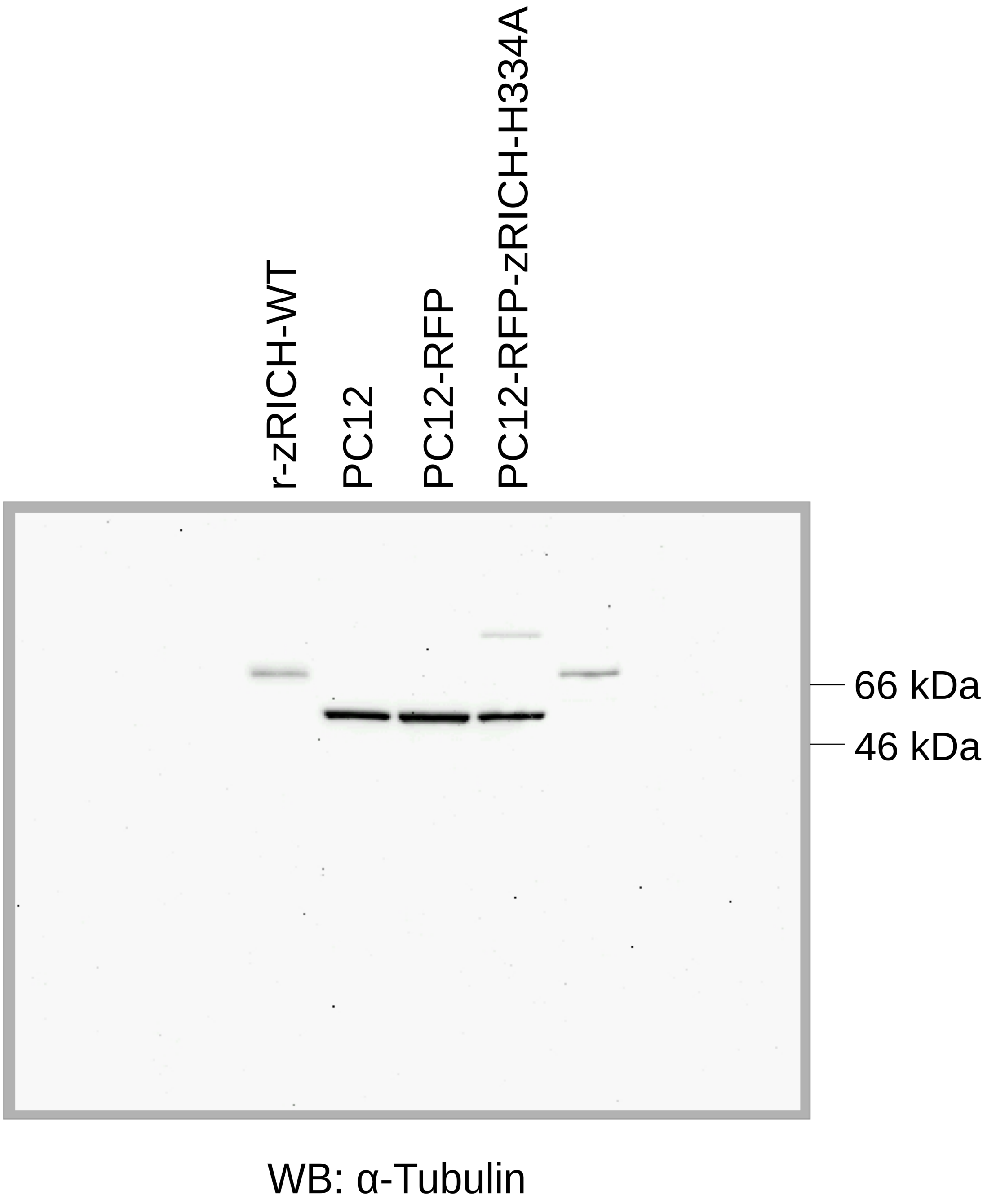

Western blot - Membrane 1 - anti-Tubulin. The figure was prepared with the original unprocessed (uncropped) image obtained from the Kodak 440 Imager Station. A box filled with grey color was used as background to facilitate the identification of the edges of the image. Please see Figure 1 for comparison and detailed description.

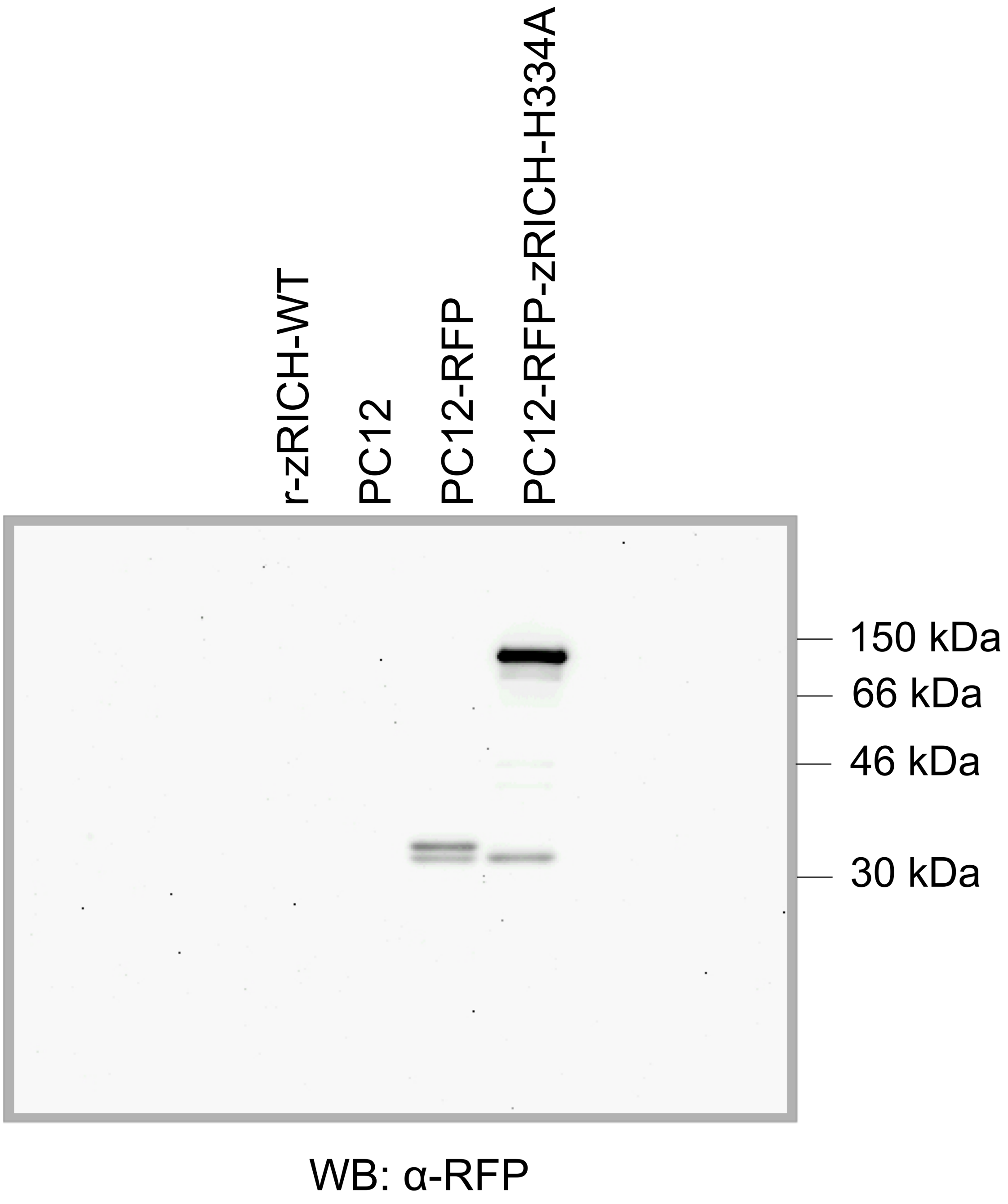

Western blot - Membrane 2 - anti-RFP. The figure was prepared with the original unprocessed (uncropped) image obtained from the Kodak 440 Imager Station. A box filled with grey color was used as background to facilitate the identification of the edges of the image. Please see Figure 1 for comparison and detailed description.

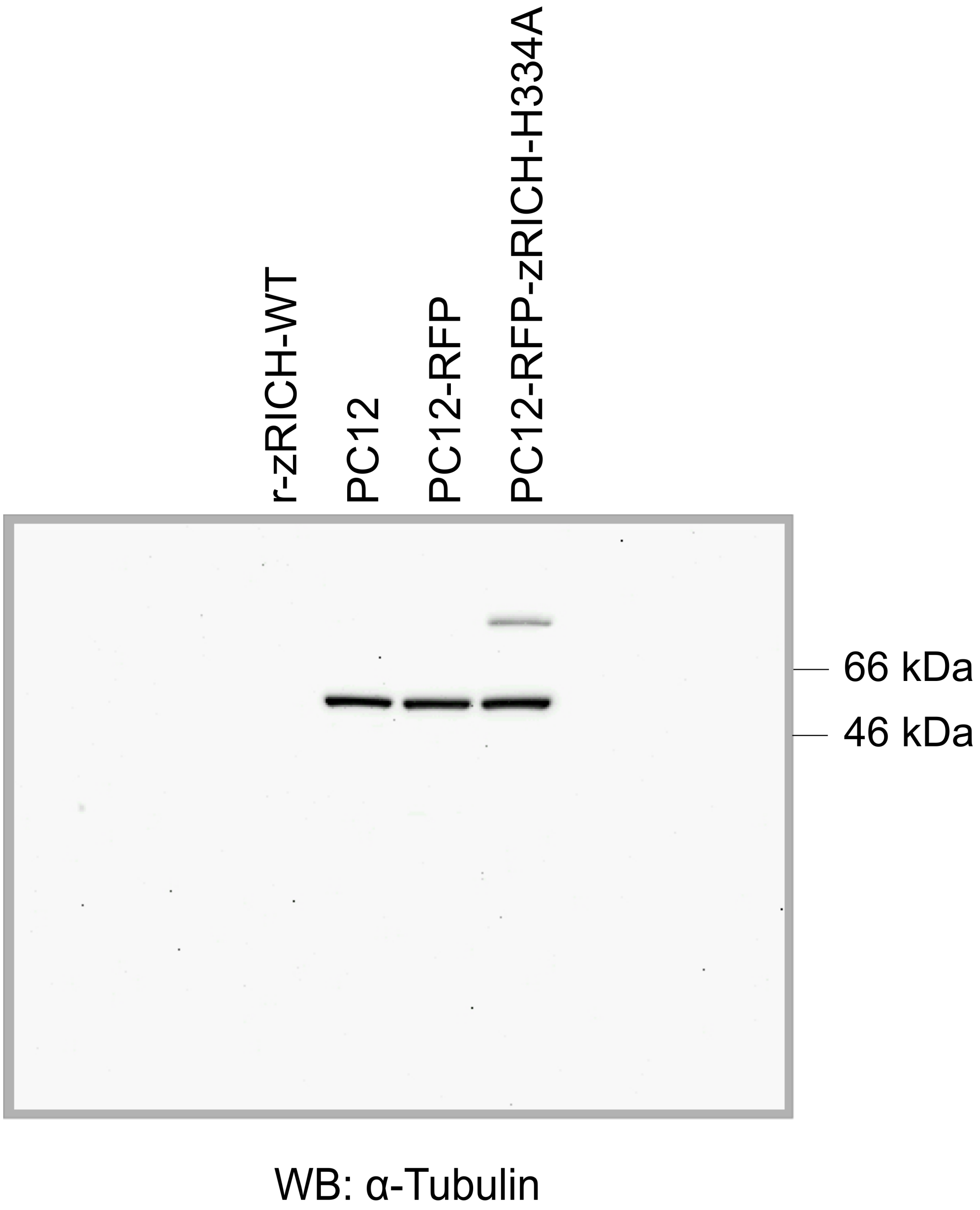

Western blot - Membrane 2 - anti-Tubulin. The figure was prepared with the original unprocessed (uncropped) image obtained from the Kodak 440 Imager Station. A box filled with grey color was used as background to facilitate the identification of the edges of the image. Please see Figure 1 for comparison and detailed description.
